# Three New Depsipeptides, Homiamides A–C, Isolated from *Streptomyces* sp., ROA-065

**DOI:** 10.3390/molecules29235539

**Published:** 2024-11-23

**Authors:** Jeong-Hyeon Kim, Ji Young Lee, Juri Lee, Prima F. Hillman, Jihye Lee, Byeongchan Choi, Man-Jeong Paik, Songyi Lee, Sang-Jip Nam

**Affiliations:** 1 Department of Chemistry and Nanoscience, Ewha Womans University, Seoul 03760, Republic of Korea; sub03101@gmail.com (J.-H.K.); ljr9703@gmail.com (J.L.); 2 Institute of Sustainable Earth and Environmental Dynamics (SEED), Pukyong National University, 365 Sinseon-ro, Nam-gu, Busan 48547, Republic of Korea; jji123789@naver.com; 3 Department of Chemistry, Faculty of Mathematics and Natural Sciences, Universitas Andalas, Kampus Limau Manis, Padang 25163, Indonesia; prima.fitria@sci.unand.ac.id; 4 Laboratories of Marine New Drugs, Redone Technologies Co., Ltd., Jangseong-gun 57247, Republic of Korea; ljh@urc.kr; 5 College of Pharmacy, Sunchon National University, Suncheon 57922, Republic of Korea; chlqudcks456@naver.com (B.C.); paik815@scnu.ac.kr (M.-J.P.); 6 Department of Chemistry, Pukyong National University, Busan 48513, Republic of Korea; 7 Industry 4.0 Convergence Bionics Engineering, Pukyong National University, Busan 48513, Republic of Korea

**Keywords:** *Streptomyces*, depsipeptide, antibacterial activity, Marfey’s method, gas chromatography mass spectrometry

## Abstract

Three new depsipeptides, homiamides A–C (**1**–**3**), were isolated from a marine sediment-derived strain of *Streptomyces* sp., ROA-065. The planar structures of homiamides A–C (**1**–**3**) were elucidated using mass spectrometry (MS) and nuclear magnetic resonance (NMR) spectroscopic data. The absolute configurations of **1**–**3** were deduced from the application of the Marfey’s method and GC-MS analysis after formation of the *O*-trifluoroacetylated (*S*)-(+)-methyl-2-butyl ester derivatives of amino acids. Compounds **1**–**3** exhibited weak anti-bacterial activities against both Gram-positive bacteria and Gram-negative bacteria, with compound **1** showing MIC values of 32–64 μg/mL. In antifouling assays, compounds **1** and **2** displayed moderate activity against *Micrococcus luteus* KCTC 3063, while compound **3** exhibited weak activity against all tested bacteria.

## 1. Introduction

Marine-derived actinomycetes, particularly those from the *Streptomyces* genus, have emerged as a valuable source of structurally diverse secondary metabolites with promising therapeutic applications [1,2,3,4]. Given the increasing threat of multidrug-resistant pathogens and the limitations of conventional drug discovery pipelines, marine-derived actinomycetes offer an attractive alternative for the identification of novel bioactive compounds [5].

The *Streptomyces* genus, renowned for its prolific production of antibiotics and other bioactive compounds in terrestrial environments, has demonstrated similar potential in marine ecosystems. Adapted to the unique conditions of marine environments, marine *Streptomyces* species have been found to produce metabolites with distinct chemical structures and potent biological activities [6,7]. Consequently, *Streptomyces* are a promising focus for drug discovery, particularly in the development of novel antibiotics. Moreover, their widespread presence in diverse marine sources, such as fish, mollusks, sponges, seaweeds, seawater and marine sediments, highlights their potential as a rich resource for bioprospecting [8,9,10,11].

Among the bioactive compounds produced by marine *Streptomyces* are depsipeptides, which possess both peptide and ester bonds. These compounds have garnered attention for their unique chemical structures and broad pharmacological properties, including anticancer, antimicrobial, and antiviral activities [12,13,14]. For example, valinomycin, isolated from *Streptomyces griseus*, cavomycins A–C, discovered from *Streptomyces cavourensis*, and streptocinnamides A and B, derived from *Streptomyces* sp. KMM 9044, are representative marine-derived depsipeptides with promising antibiotic properties [15,16,17].

**Figure 1:**
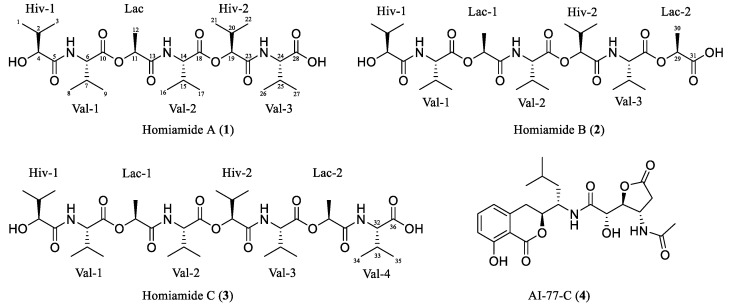
Chemical structures of compounds **1**–**4**.

In our ongoing search for bioactive natural products from marine-derived microorganisms, we identified *Streptomyces* sp. ROA-065, isolated from marine sediment collected at a depth of 30 m off the coast of Pohang using a remotely operated vehicle (ROV). Investigation of the chemical components of the culture broth from this strain has led to the isolation of three depsipeptides, homiamides A–C (**1**–**3**) (Figure 1).

## 2. Results and Discussion

*Homiamide A* (**1**) was isolated as a colorless amorphous solid, and its molecular formula was confirmed as C_28_H_49_N_3_O_10_ based on HR-FAB-MS data of the sodium-adducted molecule [M + Na]^+^ at *m*/*z* 610.3318 (calcd for C_28_H_49_N_3_O_10_Na^+^, 610.3316).

The ^1^H NMR spectrum for compound **1** exhibited three exchangeable amide protons (*δ*_H_ 7.43, 7.45, and 8.07), six *α*-protons (*δ*_H_ 3.93, 4.00, 4.04, 4.44, 4.93, and 5.25), five *β*-protons (*δ*_H_ 2.09, 2.11, 2.28, 2.35, and 2.35), and overlapped methyls (*δ*_H_ 0.88, 0.95, 0.96, 0.99, 1.00, 1.01 (×2), 1.02, 1.06 (×2), and 1.46, Table 1). The ^13^C NMR and HSQC spectroscopic data displayed 28 carbon signals, including six ester or amide carbonyl carbons (*δ*_C_ 171.2, 172.7, 172.9, 173.9, 174.5, and 176.4), six *α*-carbons (*δ*_C_ 57.7, 59.7, 60.6, 70.9, 76.0, and 79.8), five *β*-carbons (*δ*_C_ 29.0, 29.5, 29.7, 30.2, and 31.5), and eleven methyl carbons (*δ*_C_ 16.2, 16.8, 17.6, 18.4, 18.9, 19.1, 19.2, 19.4, 19.5, 19.6, and 20.0, Table 1).

**Figure 2:**
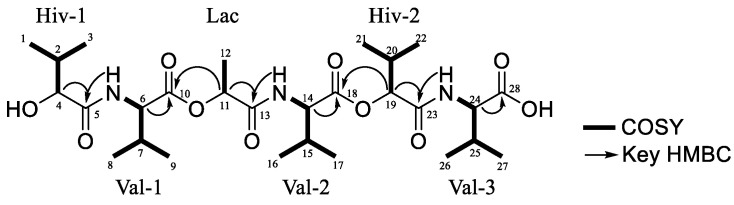
COSY and key HMBC correlations of **1**.

Detailed analysis of 2D NMR spectroscopic data enabled the construction of the structure, revealing six distinct fragments: two 2-hydroxyisovaleric acids (Hiv), three valines (Val), and one lactic acid (Lac). The two Hiv moieties were assigned using COSY and HMBC correlations. The ^1^H-^1^H COSY cross-peaks (H-1/H-2/H-3/H-4) and long-range HMBC correlations from H-4 to C-5 permitted assignment of the Hiv-1 moiety, while the ^1^H-^1^H COSY cross-peaks (H-19/H-20/H-21/H-22) and long-range HMBC correlations from H-19 to C-23 provided the assignment of the Hiv-2 moiety. The three valine units were similarly determined through COSY and HMBC correlations. The ^1^H-^1^H COSY cross-peaks (H-6/H-7/H-8/H-9) and long-range HMBC correlations from H-6 to C-10, the ^1^H-^1^H COSY cross-peaks (H-14/H-15/H-16/H-17) and long-range HMBC correlations from H-14 to C-18, and the ^1^H-^1^H COSY cross-peaks (H-24/H-25/H-26/H-27) and long-range HMBC correlations from H-24 to C-28 permitted assignment of the Val-1, Val-2, and Val-3 moiety, respectively. The Lac moiety was deduced from the COSY cross-peak (H-11/H-12) and HMBC correlations from H-11 to C-13. The sequence of amino acid fragments was established using key long-range HMBC spectroscopic data analysis. The HMBC correlations from 6-NH to C-5, from H-11 to C-10, from 14-NH to C-13, from H-19 to C-18, and from 24-NH to C-23 permitted the assignment of a fragment sequence of Hiv-Val-Lac-Val-Hiv-Val, which allowed the completion of the planar structure of **1** (Figure 2).

*Homiamide B* (**2**) was isolated as a colorless amorphous solid, with a molecular formula of C_31_H_53_N_3_O_12_ based on HR-FAB-MS data of the sodium-adducted molecule [M + Na]^+^ at *m*/*z* 682.3524 (calcd for C_31_H_53_N_3_O_12_Na^+^, 682.3527).

The ^1^H NMR spectrum of **2** exhibited three exchangeable amide protons (*δ*_H_ 7.34, 7.58, and 7.90), seven *α*-protons (*δ*_H_ 3.91, 4.00, 4.04, 4.33, 4.90, 4.97, and 5.12), five *β*-protons (*δ*_H_ 2.04, 2.12, 2.25, 2.27, and 2.35), and overlapped methyl doublet protons (*δ*_H_ 0.90, 0.96, 0.97 (×2), 0.98 (×2), 1.00, 1.02, 1.03, 1.08, 1.49, and 1.50, Table 1). The ^13^C NMR and HSQC spectroscopic data displayed 31 carbon signals, including seven ester or amide carbonyl carbons (*δ*_C_ 170.5, 172.2, 173.1, 173.8, 174.8, 174.9, and 177.1), seven *α*-carbons (*δ*_C_ 58.4, 59.5, 60.8, 70.4, 71.6, 76.7, and 80.0), five *β*-carbons (*δ*_C_ 28.9, 29.3, 29.6, 30.1, and 31.9), and twelve methyl carbons (*δ*_C_ 16.6, 16.8 (×2), 17.8, 18.8, 19.1 (×2), 19.2, 19.4, 19.5, 19.6, and 19.8, Table 1).

**Figure 3:**
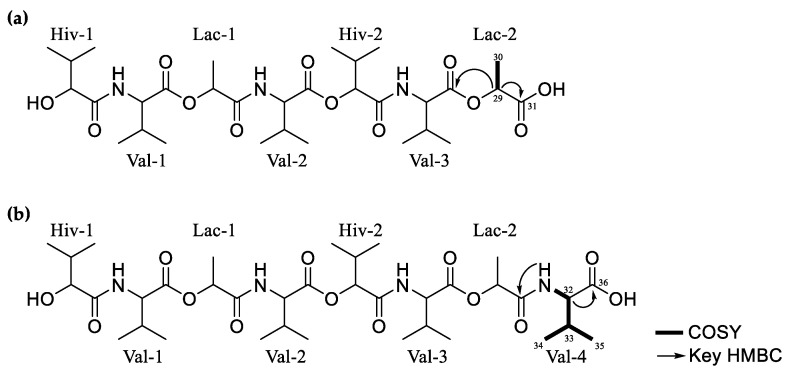
(**a**) COSY and key HMBC correlations of **2**; (**b**) COSY and key HMBC correlations of **3**.

Analysis of the 2D NMR spectroscopic data for **2** revealed a structure similar to that of **1**, with two Hiv, three Val, and two Lac residues. An additional Lac moiety was determined by a COSY correlation of H-29/H-30 and a HMBC correlation from H-29 to C-31. Key HMBC correlations from 6-NH to C-5, from H-11 to C-10, from 14-NH to C-13, from H-19 to C-18, from 24-NH to C-23, and from H-29 to C-28 established the sequence Hiv-1/Val-1/Lac-1/Val-2/Hiv-2/Val-3/Lac-2, completing the planar structure of **2** (Figure 3a).

*Homiamide C* (**3**) was isolated as a colorless amorphous solid, and its molecular formula was assigned as C_36_H_62_N_4_O_13_ based on HR-FAB-MS data of the sodium-adducted molecule [M + Na]^+^ at *m*/*z* 781.4221 (calcd for C_36_H_62_N_4_O_13_Na^+^, 781.4211).

The ^1^H NMR spectrum for compound **3** displayed four exchangeable amide protons (*δ*_H_ 7.21, 7.50, 7.71, and 7.74), eight *α*-protons (*δ*_H_ 3.99, 4.05, 4.07, 4.21, 4.37, 4.75, 4.88, and 5.18), six *β*-protons (*δ*_H_ 2.08, 2.23, 2.25, 2.26, 2.29, and 2.35), and overlapping methyl doublet protons (*δ*_H_ 0.96, 0.99 (×2), 1.00, 1.01 (×2), 1.02 (×2), 1.05, 1.07, 1.10 (×2), 1.39, and 1.49, Table 1). The ^13^C NMR and HSQC spectroscopic data revealed 36 carbon signals, including eight ester or amide carbonyl carbons (*δ*_C_ 170.5, 171.7, 172.2, 172.7, 173.4, 174.1, 174.8, and 178.2), eight *α*-carbons (*δ*_C_ 58.1, 59.6, 60.6, 60.7, 68.6, 71.3, 80.0, and 80.2), six *β*-carbons (*δ*_C_ 28.7, 29.0, 29.5, 29.5, 30.2, and 30.2), and fourteen methyl carbons (*δ*_C_ 16.7, 17.2, 17.8, 18.8, 19.0, 19.2, 19.3 (×2), 19.5, 19.7 (×2), 19.8 (×2), and 20.6, Table 1).

Analysis of the 2D NMR spectroscopic data of **3** indicated that the structure of **3** closely resembled that of **2**, featuring two Hiv, four Val, and two Lac units. Further 2D NMR spectroscopic data analysis for **3** revealed the presence of an additional Val residue. This was determined by COSY correlations 32-NH/H-32/H-33/H-34/H-35, along with HMBC correlations from H-32 to C-36. The sequence of amino acid fragments in **3** was established by key long-range HMBC correlations from 6-NH to C-5, from H-11 to C-10, from 14-NH to C-13, from H-19 to C-18, from 24-NH to C-23, from H-29 to C-28, and from 32-NH to C-31.This allowed the assignment of a fragment sequence as Hiv-1/Val-1/Lac-1/Val-2/Hiv-2/Val-3/Lac-2/Val-4, completing the structural elucidation of **3** (Figure 3b).

The absolute configurations of the homiamides were determined through a combination of spectroscopic analyses and chemical methods. We suggested that homiamides A–C (**1**–**3**) possessed identical absolute configurations based on their simultaneous isolation and similar NMR data.

**Scheme 1:**
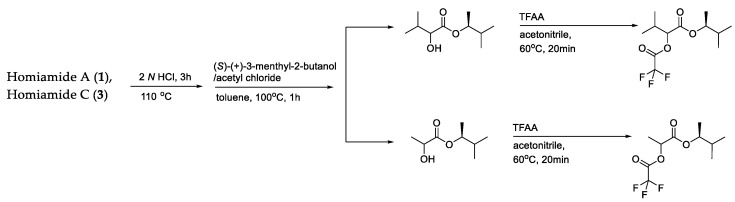
Reaction sequence for the determination of the absolute configurations of Hiv and Lac.

To determine the absolute configuration of amino acids in **1**–**3**, Marfey’s method was employed. The retention times of l- and d-FDLA derivatives of the constituent amino acids were monitored using LC-ESI-MS. The l-FDLA derivatives of the amino acids, Val, in **1**–**3** exhibited shorter retention times on the C18 HPLC column compared to their d-FDLA counterparts, indicating that the valine residues in **1**–**3** have an _L_-configuration (Figure S26). The absolute configuration of Hiv was determined as *S* via GC-MS analysis, following derivatization with *O*-trifluoroacetylated (*S*)-(+)-3-methyl-2-butyl ester (Scheme 1). The retention time of *O*-trifluoroacetylated (*S*)-(+)-3-methyl-2-butyl ester of Hiv in **1** and **3** was 14.42 min, whereas the *O*-trifluoroacetylated (*S*)-(+)-3-methyl-2-butyl ester of authentic *R*-Hiv and *S*-Hiv were eluted by GC-MS analysis at 14.24 and 14.42 min, respectively. Similarly, the absolute configuration of lactic acid was determined using the same method via GC-MS analysis after derivatization. The peak of *O*-trifluoroacetylated (*S*)-(+)-3-methyl-2-butyl ester of Lac in **1** and **3** was observed at 9.98 min in GC-MS. The *O*-trifluoroacetylated (*S*)-(+)-methyl-2-butyl ester derivatives of authentic *R*-Lac and *S*-Lac were detected at 9.66 min and 9.98 min, respectively (Figures S27–S29) [18]. Therefore, the sequence of amino residues in compounds **1**–**3** were assigned as (*S*)-Hiv-1-l-Val-1-(*S*)-Lac-l-Val-2-(*S*)-Hiv-2-l-Val-3 for **1**, (*S*)-Hiv-1-l-Val-1-(*S*)-Lac-l-Val-2-(*S*)-Hiv-2-l-Val-3-(*S*)-Lac-2 for **2**, and (*S*)-Hiv-1-l-Val-1-(*S*)-Lac-1-l-Val-2-(*S*)-Hiv-2-l-Val-3-(*S*)-Lac-2-l-Val-4 for **3**, respectively.

Additionally, a known compound, AI-77-C (**4**), was isolated and identified by comparing its NMR and MS data with previously reported ones. Compound **4** was originally isolated from *Bacillus* species and was found to exhibit ABTS^+^ radical scavenging activity, antioxidant effects, and antiulcerogenic properties [19,20].

Homiamides A–C (**1**–**3**) contain a core acid sequence of Hiv-Val-Lac-Val, which is structurally similar to the identified linear depsipeptides, cavomycins A–C, and the cyclic depsipeptide valinomycin. However, cavomycins consist of two repeated units of the Hiv-Val-Lac-Val motif with additional amino acids attached, while valinomycin is composed of three repeated units of this motif. In contrast, homiamide A has a single Hiv-Val-Lac-Val unit, with additional Hiv and Val amino acids attached. Homiamide B features the same unit with additional Hiv, Val, and Lac residues, while homiamide C is composed of two repeated Hiv-Val-Lac-Val units. Notably, homiamide C (**3**) exhibits a linear desipeptide structure, although it shares the same amino acid sequence as the cyclic octadepsipeptide, montanastatin [21].

Since homiamides represent shortened versions of cavomycins and valinomycin, it was anticipated that these compounds might also be present in the extract of the ROA-065 strain. However, LC-MS analysis of the ROA-065 extract did not detect either cavomycins or valinomycin. The absence of these compounds suggests that the thioesterase (TE) domain at the C-terminus of the last NRPS in ROA-065 is involved in product release through hydrolysis, without promoting the complex macrocyclization observed in valinomycin biosynthesis. In valinomycin-producing bacteria, the TE domain facilitates cyclization [22]. By contrast, in the case of homiamides, the linear structure suggests that the TE domain in ROA-065 lacks cyclization activity.

Compounds **1**–**3** were tested for antibacterial activity against three Gram-positive bacteria (*Bacillus subtilis* KCTC 1021, *Kocuria rhizophila* KCTC 1915, and *Staphylococcus aureus* KCTC 1621) and three Gram-negative bacteria (*Escherichia coli* KCTC 2441, *Salmonella typhimurium* KCTC 2515, and *Klebsiella pneumoniae* KCTC 2690). Compound **1** exhibited weak antibacterial activity against both Gram-positive *B. subtilis* KCTC 1021 and Gram-negative *E. coli* KCTC 2441, with a minimum inhibitory concentration (MIC) of 64 μg/mL. In addition, **1** showed weak activity against Gram-positive *K. rhizophila* KCTC 1915 and *S. aureus* KCTC 1621 and against Gram-negative *S. typhimurium* KCTC 2515 and *K. pneumonia* KCTC 2690, with a MIC of 32 μg/mL. Compounds **2** and **3** also displayed weak antibacterial activity against both tested Gram-positive bacteria and Gram-negative bacteria (Table 2).

Compounds **1**–**3** were further tested for antifouling activity against three Gram-positive bacteria (*B. subtilis* KCTC 1021, *M. luteus* KCTC 3063, and *S. aureus* KCTC 1621) and two Gram-negative bacteria (*E. coli* KCTC 2441, and *Pseudomonas fluorescens* KCTC 42821). Compound **3** showed weak activity against all five pathogenic bacteria. Compounds **1** and **2** were moderately effective against Gram-positive *M. luteus* KCTC 3063, but displayed weak activity against the other pathogens (Table 3).

## 3. Materials and Methods

### 3.1. General Experimental Procedures

The UV spectra were recorded on a Scinco UVS2100 spectrophotometer (Scinco, Daejeon, Republic of Korea) in methanol (MeOH). Optical rotations were measured using a Kruss Optronic P-8000 polarimeter (Krüss Optronic, Hamburg, Germany) with a 5 cm cell, and infrared spectra were measured with a Varian Scimitar Series FT-IR (Varian Inc., Palo Alt, Australia). HR-FAB-MS spectra were recorded on a JEOL JMS-AX505WA mass spectrometer (JEOL Ltd., Tokyo, Japan) at Seoul National University. NMR spectra were acquired with a JEOL NMR spectrometer (500 MHz for ^1^H and 125 MHz for ^13^C, JEOL Ltd., Tokyo, Japan) at Ewha Womans University, and with an Agilent 400-MR DD2 NMR spectrometer (400 MHz for ^1^H and 100 MHz for ^13^C, Agilent Technologies, Santa Clara, CA, USA) at the Ewha Drug Development Research Core Center. The measurements were performed in deuterated chloroform (CDCl_3_) at 298 K (room temperature). Chemical shifts (*δ*) are reported in ppm and coupling constants (*J*) are given in Hz. The spectra were calibrated using the residual solvent peak (*δ*_H_ 7.26 ppm and *δ*_C_ 77.2 ppm). Low resolution LC/MS data were collected using an Agilent Technologies 1260 quadrupole LC/MS system (Agilent Technologies, Santa Clara, CA, USA) and a Waters Alliance Micromass ZQ LC-MS system (Waters Corp., Milford, MA, USA) at the National Research Facilities and Equipment Center (NanoBioEnergy Materials Center) at Ewha Womans University. Analyses were performed using a reversed-phase column (Phenomenex Luna C18 (2) 100 Å, 50 mm × 4.6 mm, 5 µm) (Phenomenex Inc., Torrance, CA, USA) at a flow rate of 1.0 mL/min. Fractions were purified by semi-preparative HPLC using a Waters 996 Photodiode Array Detector HPLC (Waters Corp., Milford, MA, USA) in reversed phase column (Phenomenex Luna C18 (2) 100 Å, 250 nm × 10 mm, 5 µm) at a flow rate of 2.0 mL/min.

### 3.2. Strain Isolation

The marine-derived *Streptomyces*, strain ROA-065, was isolated from marine sediment collected off the coast of Pohang, South Korea. Strain ROA-065 was identified as the *Streptomyces* sp., showing 99.9% sequence identity to *Streptomyces cavourensis* based on NCBI blast analysis of the 16S rRNA gene. The DNA sequence of strain ROA-065 was deposited in GenBank (accession number OR775505).

### 3.3. Fermentation, Extraction, and Purification

The strain ROA-065 was cultured in an Ultra Yield Flask containing 1 L of SYP SW medium (10 g/L of soluble starch, 2 g/L of yeast extract, 4 g/L of peptone, 34.75 g/L of sea salt in 1 L of distilled water) with shaking at 120 rpm and 27 °C. After 7 days, the culture was extracted with ethyl acetate (EtOAc, 80 L total). The EtOAc-soluble fraction was concentrated in vacuo to yield 5.7 g of crude extract. A portion of the crude extract (5.5 g) was separated to conduct C18 open column chromatography, eluting with H_2_O/CH_3_OH (80:20–0:100, each of 500 mL) to obtain eight subfractions (F1 to F8). The fifth fraction (392.2 mg) was purified by a reversed phase HPLC [Phenomenex Luna C18 (2) 100 Å, (250 nm × 10 mm, 5 μm)] using an isocratic solvent system of 70% aqueous CH_3_CN to obtain 6.5 mg of homiamide A (**1**, *t*_R_ = 24.8 min). To obtain 3.5 mg of homiamide B (**2**, *t*_R_ = 43.9 min), and 3.3 mg of homiamide C (**3**, *t*_R_ = 47.4 min), the fifth fraction was further purified by reversed-phase HPLC [Phenomenex Luna C18 (2) 100 Å, (250 nm × 10 mm, 5 μm)] using an isocratic solvent system with 50% aqueous CH_3_CN.

*Homiamide A* (**1**): colorless amorphous solid;$\;{\left[\alpha \right]}_{D}^{21}$ −20.6 (*c* 0.8, MeOH); UV (MeOH) λmax (log ε) 203 (3.8) nm; IR (KBr) νmax 3307, 2967, 1666, 1201, 757 cm^−1^; ^1^H and ^13^C NMR data, see Table 1; LR-ESI-MS *m*/*z* 588.4 [M + H]^+^; HR-FAB-MS *m*/*z* 610.3318 [M + Na]^+^ (calcd for C_28_H_49_N_3_O_10_Na^+^, 610.3316).

*Homiamide B* (**2**): colorless amorphous solid; ${\left[\alpha \right]}_{D}^{21}$ −6.0 (*c* 1.0, MeOH); UV (MeOH) λmax (log ε) 203 (3.7) nm; IR (KBr) νmax 3307, 2967, 1668, 1203 757 cm^−1^; ^1^H and ^13^C NMR data, see Table 1; LR-ESI-MS *m*/*z* 682.4 [M + Na]^+^; HR-FAB-MS *m*/*z* 682.3524 [M + Na]^+^ (calcd for C_31_H_53_N_3_O_12_Na^+^, 682.3527).

*Homiamide C* (**3**): colorless amorphous solid; ${\left[\alpha \right]}_{D}^{21}\;$ +51.3 (*c* 1.0, MeOH); UV (MeOH) λmax (log ε) 203 (3.6) nm; IR (KBr) νmax 3308, 2967, 1667, 1201, 757 cm^−1^; ^1^H and ^13^C NMR data, see Table 1; LR-ESI-MS *m*/*z* 781.5 [M + Na]^+^; HR-FAB-MS *m*/*z* 781.4221 [M + Na]^+^ (calcd for C_36_H_62_N_4_O_13_Na^+^, 781.4211).

### 3.4. Homiamides A–C (***1***–***3***) Hydrolysis and Marfey’s Analysis

Homiamides A–C (**1**–**3**) were treated with 2 *N* HCl (100 μL) at 110 °C for 3 h. The hydrolysate was dried under an N_2_ gas stream and reconstituted in distilled H_2_O, then divided into two portions. After evaporating the H_2_O under the N_2_ gas stream, each sample was dissolved in 1 N NaHCO_3_ (100 μL) and derivatized with 1% d-FDLA (1-fluoro-2,4-dinitrophenyl-5-d-leucine amide) in acetone (100 μL). The reaction mixtures were heated at 60 °C for 1 h, then quenched with 2 N HCl. Acetonitrile (100 μL) was added to each product, which was then injected into an LC-ESI-MS system under the following chromatographic conditions: Phenomenex Luna C18 (2) 100 Å, 50 mm × 4.6 mm, 5 μm, at a flow rate of 1.0 mL/min. The solvent gradient employed solvent A (0.01% formic acid in H_2_O) and solvent B (0.01% formic acid in CH_3_CN), starting from A/B = 95:5 and progressing to 50:50 (50 min), 100:0 (70 min), 0:100 (85 min), and returning to 95:5 (90 min). Standard amino acids were prepared according to established protocols and analyzed under the same conditions. The reaction products were detected using UV absorption at 340 nm in positive ESI-MS mode [23].

### 3.5. Preparation of Standards for GC-MS Analysis

The stock solutions of (*R*)-and (*S*)-Lac and Hiv were prepared at 10 μg/μL in methanol. Standard working solutions of Lac and Hiv enantiomer were prepared as 1.0 μg/μL in methanol, respectively. All the standard solutions were stored at 4 °C.

### 3.6. Diastereomeric (S)-(+)-3-Methyl-2-Butylation and O-Trifluoroacetylation

First, Lac and Hiv enantiomers (10 μg) were evaporated to dryness under a gentle stream of nitrogen at 40 °C. Next, toluene (20 μL), (*S*)-(+)-3-methyl-2-butanol (10 μL), and acetyl chloride (5 μL) were added to the dry residue, and then reacted for 1 h at 100 °C for conversion as diastereomeric 3-methyl-2-butylester. Next, it was evaporated to dryness under a gentle stream of nitrogen to remove excess reagent, which was reacted with trifluoroacetic anhydride (TFAA, 20 μL) in acetonitrile (10 μL) for 20 min at 60 °C for conversion as *O*-TFA derivatives. Finally, the reaction mixture was evaporated and dissolved in toluene (30 μL), and then was analyzed by GC-MS in SIM mode as per previously recorded methods [18,24].

### 3.7. Sample Preparation for Chiral Separation of Lac and Hiv in Homiamides A and C

First, an aliquot of homiamides A and C (20 μg) was evaporated to dryness under a gentle stream of nitrogen at 40 °C. Next, toluene (20 μL), (*S*)-(+)-3-methyl-2-butanol (10 μL), and acetyl chloride (5 μL) were added to the dry residue, and then reacted for 1 h at 100 °C for conversion as diastereomeric 3-methyl-2-butylester. Then, it was evaporated to dryness under a gentle stream of nitrogen to remove excess reagent, which was reacted with TFAA (20 μL) in acetonitrile (10 μL) for 20 min at 60 °C for conversion as *O*-TFA derivatives. Finally, the reaction mixture was evaporated and dissolved in toluene (30 μL), and then was analyzed by GC-MS in SIM mode.

### 3.8. Gas Chromatography-Mass Spectrometry

The *O*-trifluoroacetylated (*S*)-(+)-3-methyl-2-butyl ester was analyzed using an Agilent 7890 gas chromatograph interfaced with an Agilent 5975c mass-selective detector (70 eV, electron impact mode, Agilent Technologies, Atlanta, GA, USA) and installed with an Ultra-2 cross linked capillary column (5% phenyl-95% methylpoly-siloxane bonded phase; 25 m × 0.20 mm I.D., 0.11 μm film thickness) (Agilent Technologies, Atlanta, GA, USA). The temperatures of the injector, interface, and ion source were 260, 280, and 230 °C, respectively. The samples were introduced into the split-injection mode (10:1). The oven temperature was maintained at 60 °C (3 min), and this temperature was increased to 300 °C (5 min) at a rate of 5 °C/min.

### 3.9. Antibacterial Activity Assay

Antibacterial susceptibilities were tested against six pathogenic bacteria (*Escherichia coli* KCTC 2441, *Salmonella typhimurium* KCTC 2515, *Klebsiella pneumonia* KCTC 2690, *Bacillus subtilis* KCTC 1021, *Kocuria rhizophila* KCTC 1915, and *Staphylococcus aureus* KCTC 1621). All bacteria were cultured in Mueller–Hinton broth (MHB) overnight. They were modified to McFarland standard 0.5 (1.0 × 10^8^ cfu/mL) which is equivalent to [C] 5.0 × 10^5^ cfu/mL. The test compounds and positive controls (ampicillin and vancomycin) were dissolved in DMSO to a concentration of 10 mg/mL firstly, then diluted to 256 μg/mL in MHB broth. A total of 100 μL of each diluted sample was added to the first column of a sterile 96-well microtiter plate and serially diluted to 0.25 μg/mL. Then, 50 μL aliquots of appropriately regulated bacterial cultures were added to each well. The final concentrations tested were 128, 64, 32, 16, 8, 4, 2, 1, 0.5, and 0.25 μg/mL. The 96-well plates were then incubated for 18 to 24 h at 37 °C. MIC values were visually determined as the lowest concentration that inhibited bacterial growth, and absorbance at 600 nm was measured using a SpectraMax^®^ ABS Plus microplate reader (Molecular devices, San Jose, CA, USA). The experiments were repeated in triplicate.

### 3.10. Quorum Sensing Inhibition Assay

The quorum sensing inhibition activities of compounds **1** to **3** were evaluated in vitro using a modified MIC assay. *Bacillus subtilis* KCTC 1021 was cultured in Luria Bertani Broth (LB), *Micrococcus luteus* KCTC 3063 was cultured in Marine Broth (MB), *Staphylococcus aureus* KCTC 1621 and *Escherichia coli* KCTC 2441 were cultured in Tryptic Soy Broth (TSB), and *Pseudomonas fluorescens* KCTC 42821 was cultured in King’s Broth (KB) at 37 °C for 24 h. Rifampin and kanamycin were used as positive controls. The experiment was carried out in triplicate.

## Figures and Tables

**Table 1:** NMR data for **1**–**3** in chloroform-*d*.

Unit	No.	Homiamide A (1) ^a^				Homiamide B (2) ^a^		Homiamide C (3) ^a^	
		*δ*_C_, Type	*δ*_H_, (*J* in Hz) ^b^	COSY	HMBC	*δ*_C_, Type	*δ*_H_, (*J* in Hz) ^b^	*δ*_C_, Type	*δ*_H_, (*J* in Hz) ^b^
Hiv-1	1	16.2, CH_3_	0.88, d (6.9)			16.8, CH_3_	0.90, d (6.8)	16.7, CH_3_	1.01, d (6.9) ^c^
	2	31.5, CH	2.11, m	1, 3		31.9, CH	2.04, m	30.2, CH	2.35, m
	3	18.9 ^c^, CH_3_	1.02, d (6.9) ^c^			18.8 ^c^, CH_3_	1.02, d (6.8) ^c^	19.0 ^c^, CH_3_	1.10, d (6.9) ^c^
	4	76.0, CH	3.93, d (4.3)	2	5	76.7, CH	3.91, d (4.3)	80.0, CH	4.88, d (3.6)
	5	176.4, C				177.1, C		170.5, C	
Val-1	6	59.7, CH	4.00, dd (5.9, 4.3)	7	10	59.5, CH	4.04, dd (5.9, 4.3)	58.1, CH	4.37, t (8.0)
	6-NH		7.43, br d (5.5)	6	5		7.58, br d (5.5)		7.21, d (9.1)
	7	29.5, CH	2.09, m	8, 9		29.3, CH	2.12, m	29.5, CH	2.29, m
	8	19.5 ^c^, CH_3_	1.06, d (6.7) ^c^			19.2 ^c^, CH_3_	0.95, d (6.7) ^c^	19.3 ^c^, CH_3_	1.01, d (6.7) ^c^
	9	19.1 ^c^, CH_3_	1.01, d (6.7) ^c^			19.1 ^c^, CH_3_	1.00, d (6.7) ^c^	19.2 ^c^, CH_3_	1.02, d (6.7) ^c^
	10	172.9, C				173.8, C		172.7, C	
Lac-1	11	70.9, CH	5.25, q (6.9)	12	10, 13	71.6, CH	5.12, q (6.9)	68.6, CH	4.21, q (6.9)
	12	17.6, CH_3_	1.46, d (6.9)			17.8, CH_3_	1.50, d (6.9)	20.6, CH_3_	1.39, d (6.9)
	13	172.7, C				173.1, C		178.2, C	
Val-2	14	60.6, CH	4.04, dd (6.3, 4.9)	15	18	60.8, CH	4.00, dd (6.3, 4.9)	59.6, CH	4.07, dd (6.8, 4.6)
	14-NH		8.07, br d (6.2)	14	13		7.90, br d (6.2)		7.50, d (5.5)
	15	29.0, CH	2.28, m	16, 17		28.9, CH	2.25 m	29.5, CH	2.08, m
	16	19.6 ^c^, CH_3_	0.95, d (6.7) ^c^			19.3 ^c^, CH_3_	1.03, d (6.7) ^c^	19.3 ^c^, CH_3_	1.02, d (6.7) ^c^
	17	19.4 ^c^, CH_3_	1.06, d (6.7) ^c^			19.8 ^c^, CH_3_	1.08, d (6.7) ^c^	19.5 ^c^, CH_3_	1.05, d (6.7) ^c^
	18	173.9, C				174.8, C		174.8, C	
Hiv-2	19	79.8, CH	4.93, d (3.3)	20	18, 23	80.0, CH	4.90, d (3.3)	80.2, CH	4.75, d (4.0)
	20	30.2, CH	2.35, m	21, 22		30.1, CH	2.35, m	29.0, CH	2.25, m
	21	16.8 ^c^, CH_3_	1.01, d (6.7) ^c^			16.6 ^c^, CH_3_	0.98, d (6.7) ^c^	17.2 ^c^, CH_3_	1.00, d (6.7) ^c^
	22	19.2 ^c^, CH_3_	0.96, d (6.7) ^c^			19.5 ^c^, CH_3_	0.97, d (6.7) ^c^	19.5 ^c^, CH_3_	0.97, d (6.7) ^c^
	23	171.2, C				170.5, C		171.7, C	
Val-3	24	57.7, CH	4.44, br t (8.0)	25	28	58.4, CH	4.33, br t (8.0)	60.7, CH	3.99, dd (7.3, 4.9)
	24-NH		7.45, br d (8.0)	24	23		7.34, br d (8.0)		7.71, d (5.8)
	25	29.7, CH	2.35, m	26, 27		29.6, CH	2.27, m	28.7, CH	2.23, m
	26	20.0 ^c^, CH_3_	0.99, d (6.6) ^c^			19.6 ^c^, CH_3_	0.97, d (6.6) ^c^	19.8 ^c^, CH_3_	0.96, d (6.6) ^c^
	27	18.4 ^c^, CH_3_	1.00, d (6.6) ^c^			19.1 ^c^, CH_3_	1.00, d (6.6) ^c^	19.7 ^c^, CH_3_	0.99, d (6.6) ^c^
	28	174.5, C				172.2, C		174.1, C	
Lac-2	29					70.4, CH	4.97, q (7.0)	71.3, CH	5.18, q (7.0)
	30					16.8, CH_3_	1.49, d (7.0)	17.8, CH_3_	1.49, d (7.0)
	31					174.9, C		172.2, C	
Val-4	32							60.6, CH	4.05, dd (6.8, 5.5)
	32-NH								7.74, d (5.5)
	33							30.2, CH	2.26, m
	34							19.8 ^c^, CH_3_	1.10, d (7.2) ^c^
	35							18.8 ^c^, CH_3_	1.07, d (7.2) ^c^
	36							173.4, C	

^a^ 400 MHz for ^1^H NMR and 100 MHz for ^13^C NMR. ^b^ Numbers of attached protons were determined by analysis of 2D spectra. ^c^ Signals were overlapped.

**Table 2:** Antibacterial activity (MIC, μg/mL) of compounds **1** to **3**.

Minimal Inhibitory Concentration (μg/mL)						
	Gram (+) Bacteria			Gram (−) Bacteria		
Compounds	*B. subtilis* KCTC 1021	*K. rhizophila* KCTC 1915	*S. aureus* KCTC 1621	*E. coli* KCTC 2441	*S. typhimurium* KCTC 2515	*K. pneumonia* KCTC 2690
**1**	64	32	32	64	32	32
**2**	64	32	32	64	32	64
**3**	64	32	64	64	64	32
Ampicillin	1	2	8	32	16	32
Vancomycin	0.25	0.25	4	64	32	32

**Table 3:** Quorum sensing inhibition results of compounds **1** to **3**.

Quorum Sensing (MIC, μg/mL)					
	Gram (+) Bacteria			Gram (−) Bacteria	
Compounds	*B. subtilis* KCTC 1021	*M. luteus* KCTC 3063	*S. aureus* KCTC 1621	*E. coli* KCTC 2441	*P. fluorescens* KCTC 42821
**1**	32	16	32	64	32
**2**	64	16	32	32	32
**3**	32	32	64	64	32
Rifampin	0.25	0.25	0.25	32	16
Kanamycin	4	32	1	16	16

## Data Availability

The original contributions presented in the study are included in the article/Supplementary Material, further inquiries can be directed to the corresponding authors.

## Supplementary Materials

The following supporting information can be downloaded at: https://www.mdpi.com/article/10.3390/molecules29235539/s1. Figures S1–S25: 1D, 2D NMR, HRMS and FT-IR spectra of the isolation of homiamides A–C; Figures S26–S29: LC, GC-MS analysis for absolute configurations of homiamides A–C.
