# Supplementary material for: Three New Depsipeptides, Homiamides A–C, Isolated from Streptomyces sp., ROA-065
Source: Molecules. 2024 Nov 23;29(23):5539. doi: 10.3390/molecules29235539 (PMC11643055; doi:10.3390/molecules29235539)

Supporting Information

Three New Depsipeptides, Homiamides A–C, Isolated from *Streptomyces* sp., ROA-065

Jeong-Hyeon Kim [1,†], Ji Young Lee [2,†], Juri Lee [1], Prima F. Hillman [3], Jihye Lee [4], Byeongchan Choi [5], Man-Jeong Paik [5], Songyi Lee [6,7,*] and Sang-Jip Nam [1,*]

1 Department of Chemistry and Nanoscience, Ewha Womans University, Seoul 03760, Republic of Korea; sub03101@gmail.com (J.-H.K.); ljr9703@gmail.com (J.L.)

2 Institute of Sustainable Earth and Environmental Dynamics (SEED), Pukyong National University, 365 Sinseon-ro, Nam-gu, Busan 48547, Republic of Korea; jji123789@naver.com

3 Department of Chemistry, Faculty of Mathematics and Natural Sciences, Universitas Andalas, Kampus Limau Manis, Padang 25163, Indonesia; prima.fitria@sci.unand.ac.id

4 Laboratories of Marine New Drugs, Redone Technologies Co., Ltd., Jangseong-gun 57247, Republic of Korea; ljh@urc.kr

5 College of Pharmacy, Sunchon National University, Suncheon 57922, Republic of Korea; chlqudcks456@naver.com (B.C.); paik815@scnu.ac.kr (M.-J.P.)

6 Department of Chemistry, Pukyong National University, Busan 48513, Republic of Korea

7 Industry 4.0 Convergence Bionics Engineering, Pukyong National University, Busan 48513, Republic of Korea

* Correspondence: slee@pknu.ac.kr (S.L.); sjnam@ewha.ac.kr (S.-J.N.); Tel.: +82-51-629-5592 (S.L.); +82-2-3277-6805 (S.-J.N.)

† These authors contributed equally to this work.

Table of Contents

Figure S1. ^{1}H NMR spectrum of homiamide A (**1**) in chloroform-*d* ········ 3

Figure S2. ^{13}C NMR spectrum of homiamide A (**1**) in chloroform-*d* ········ 4

Figure S3. COSY spectrum of homiamide A (**1**) in chloroform-*d* ········ 5

Figure S4. HSQC NMR spectrum of homiamide A (**1**) in chloroform-*d* ········ 6

Figure S5. HMBC NMR spectrum of homiamide A (**1**) in chloroform-*d* ········ 7

Figure S6. NOESY NMR spectrum of homiamide A (**1**) in chloroform-*d* ········ 8

Figure S7. HRMS Spectrum for homiamide A (**1**) ········ 9

Figure S8. FT-IR spectrum of homiamide A (**1**) ········ 10

Figure S9. ^{1}H NMR spectrum of homiamide B (**2**) in chloroform-*d* ········ 11

Figure S10. ^{13}C NMR spectrum of homiamide B (**2**) in chloroform-*d* ········ 12

Figure S11. COSY spectrum of homiamide B (**2**) in chloroform-*d* ········ 13

Figure S12. HSQC NMR spectrum of homiamide B (**2**) in chloroform-*d* ········ 14

Figure S13. HMBC NMR spectrum of homiamide B (**2**) in chloroform-*d* ········ 15

Figure S14. NOESY NMR spectrum of homiamide B (**2**) in chloroform-*d* ········ 16

Figure S15. HRMS spectrum for homiamide B (**2**) ········ 17

Figure S16. FT-IR spectrum for homiamide B (**2**) ········ 18

Figure S17. ^{1}H NMR spectrum of homiamide C (**3**) in chloroform-*d* ········ 19

Figure S18. ^{13}C NMR spectrum of homiamide C (**3**) in chloroform-*d* ··· 20
Figure S19. COSY spectrum of homiamide C (**3**) in chloroform-*d* ··· 21
Figure S20. HSQC NMR spectrum of homiamide C (**3**) in chloroform-*d* ··· 22
Figure S21. HMBC NMR spectrum of homiamide C (**3**) in chloroform-*d* ··· 23
Figure S22. NOESY NMR spectrum of homiamide C (**3**) in chloroform-*d* ··· 24
Figure S23. HRMS spectrum for homiamide C (**3**) ··· 25
Figure S24. FT-IR spectrum for homiamide C (**3**) ··· 26
Figure S25. ^{1}H NMR spectrum of AI-77-C (**4**) in methanol-d_4 ··· 27
Figure S26. LC chromatograms of L- and D-FDLA derivatives of Valine ··· 28
Figure S27. Chromatograms of Lac and Hiv in **(a)** homiamide A (**1**) and **(b)** homiamide C (**3**) as tri-methylsilyl derivative ··· 29
Figure S28. GC-MS analysis of *O*-trifluoroacetylated (*S*)-(+)-3-methyl-2-butyl ester of Lac and Hiv standards **(a)** SIM chromatogram **(b)** Expanded SIM chromatogram **(c)** Selected electron ionization mass spectrum of Lac and Hiv ··· 30
Figure S29. SIM Chromatograms for *O*-trifluoroacetylated (*S*)-(+)-3-methyl-2-butyl ester of Lac and Hiv enantiomers in **(a)** homiamide A (**1**) and **(b)** homiamide C (**3**) ··· 31

Figure S1. ^{1}H NMR spectrum of homiamide A (**1**) in chloroform-*d*

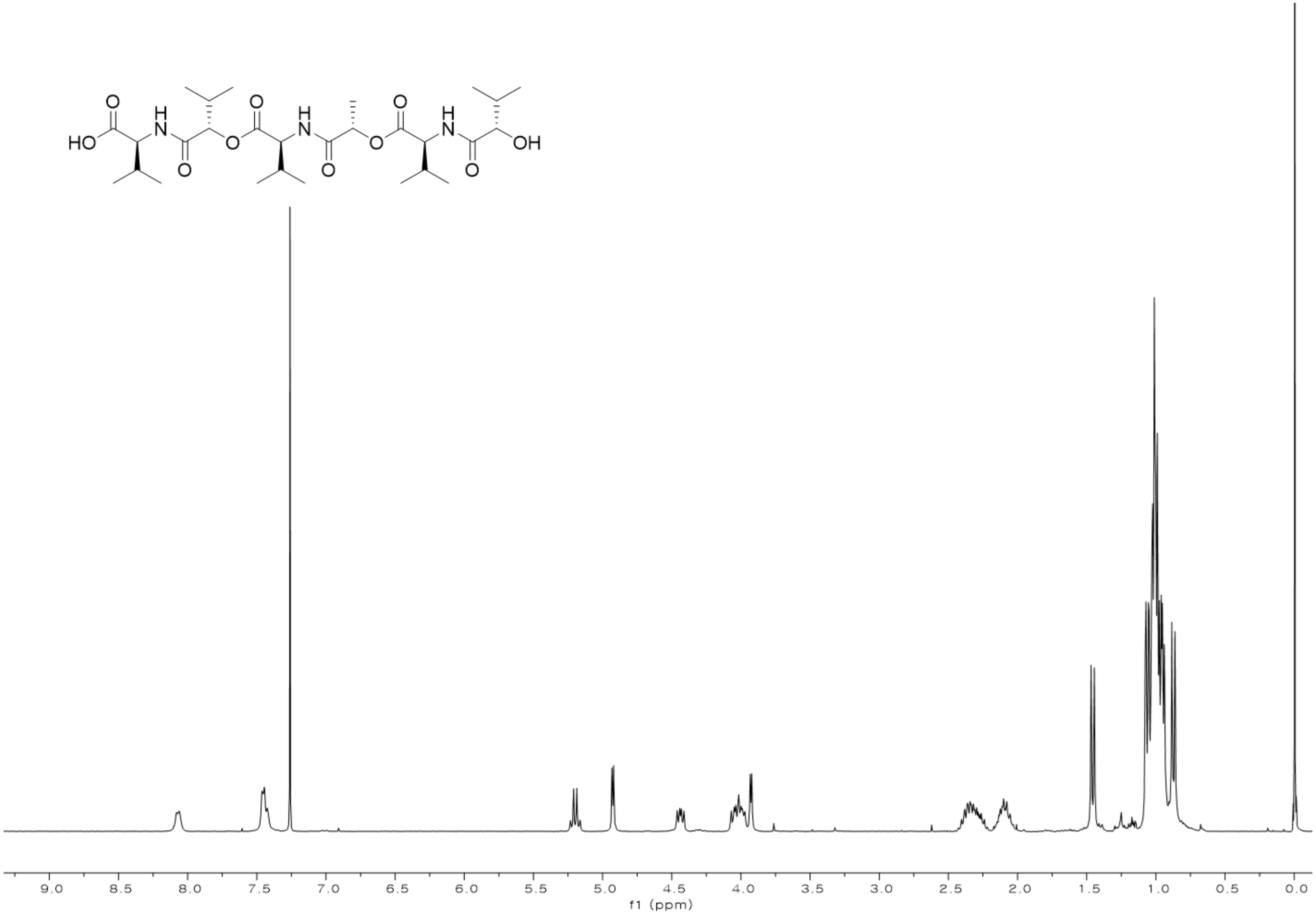

Figure S2. ^{13}C NMR spectrum of homiamide A (**1**) in chloroform-*d*

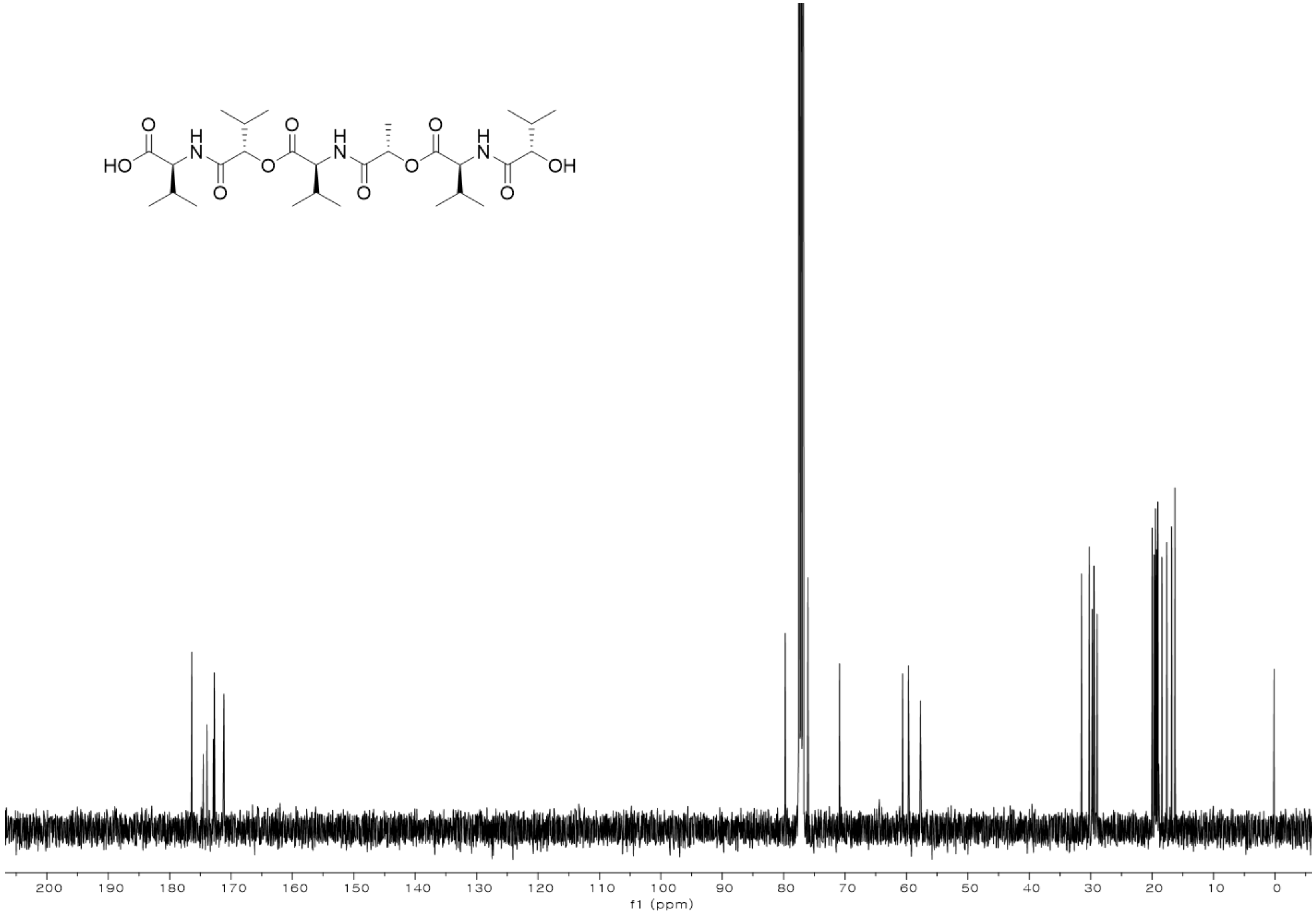

Figure S3. COSY spectrum of homiamide A (**1**) in chloroform-*d*

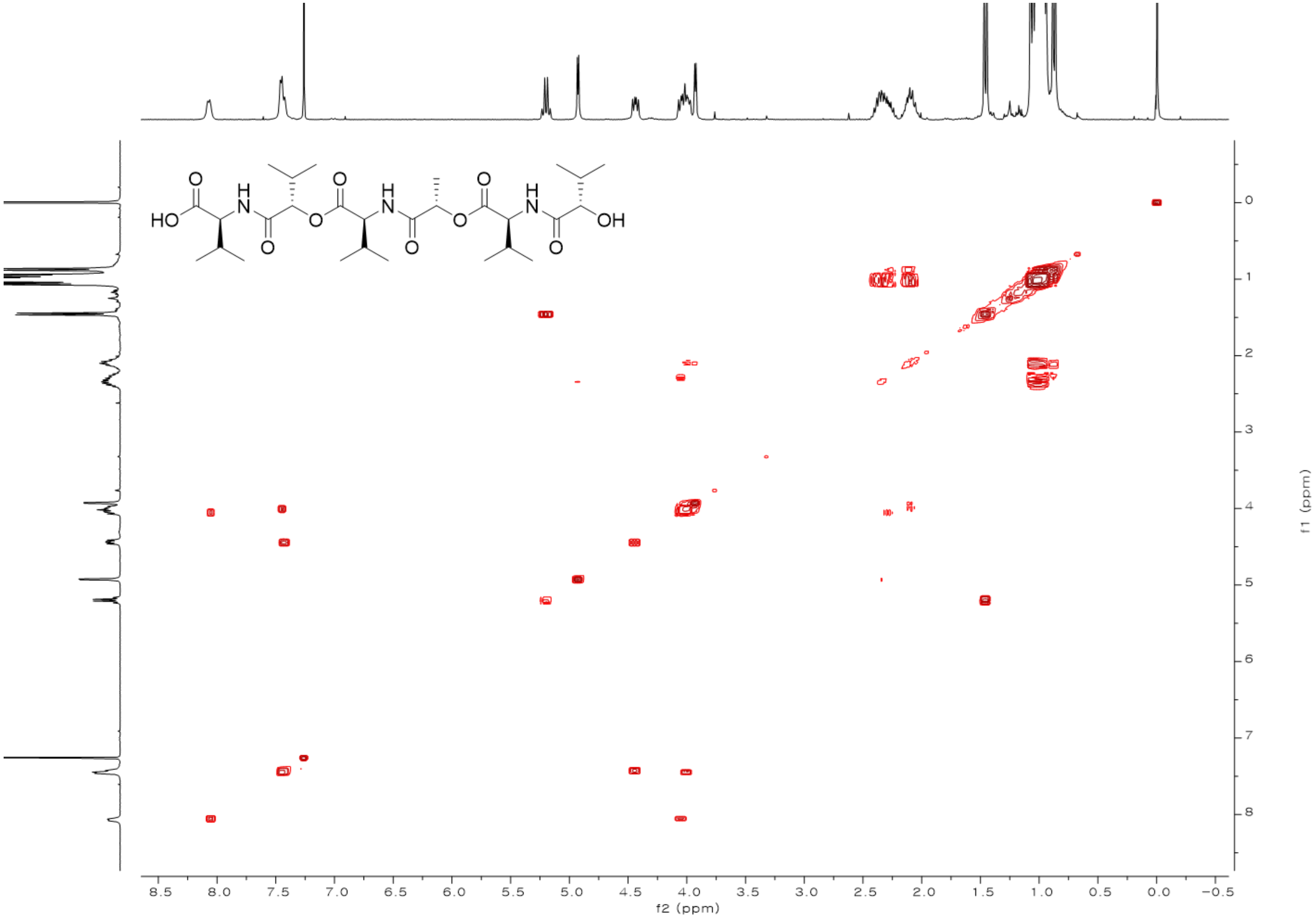

Figure S4. HSQC NMR spectrum of homiamide A (**1**) in chloroform-*d*

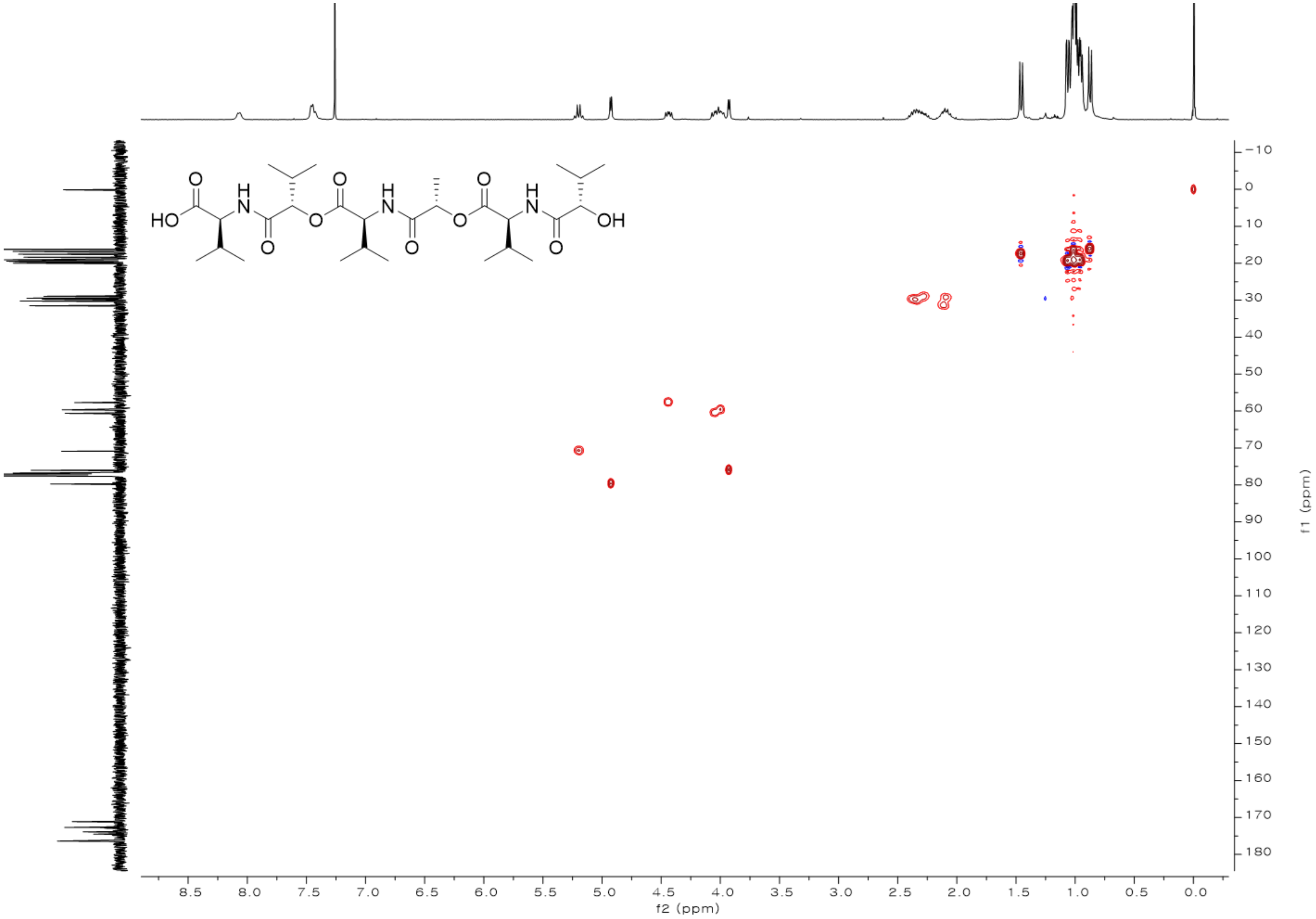

Figure S5. HMBC NMR spectrum of homiamide A (**1**) in chloroform-*d*

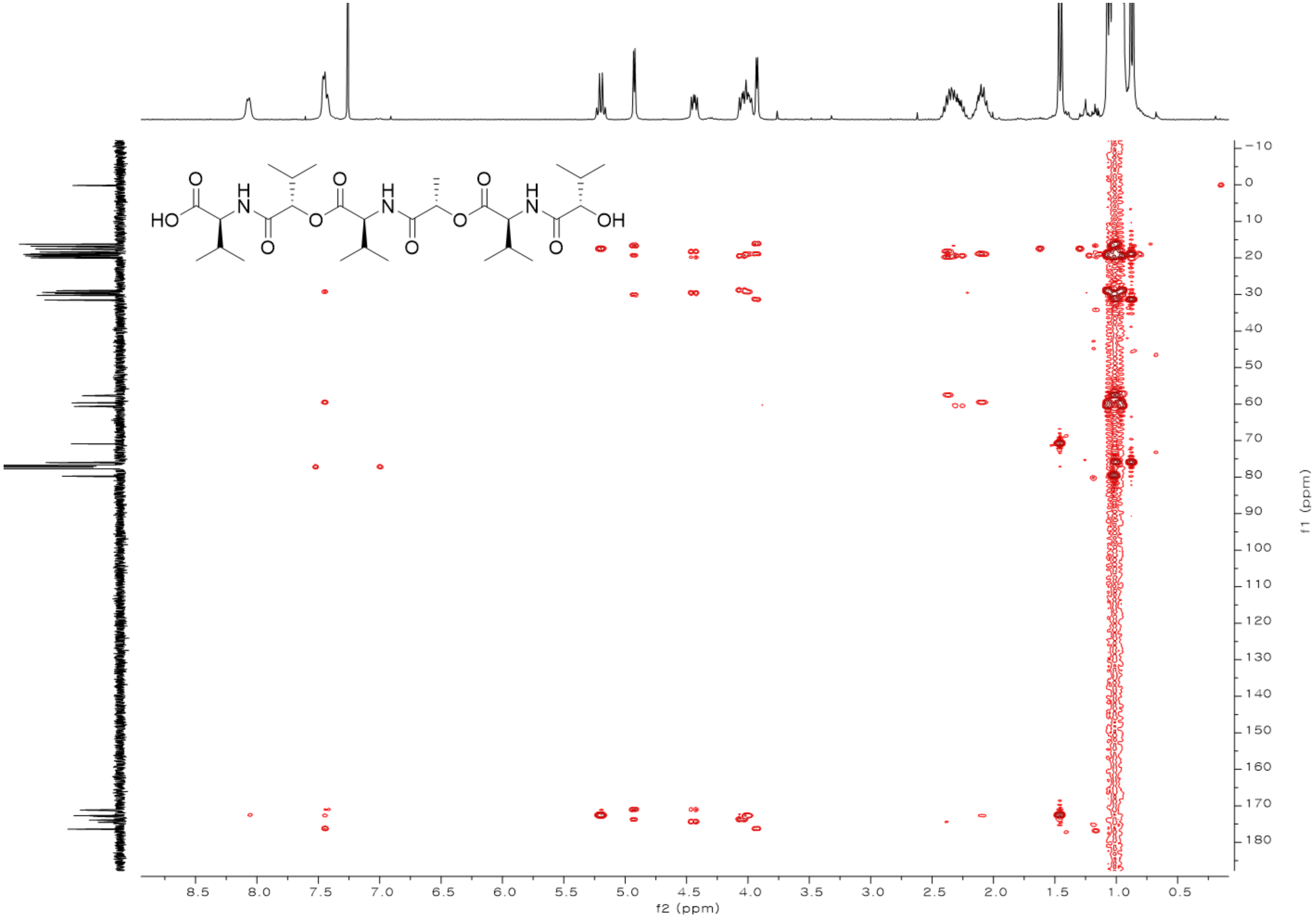

Figure S6. NOESY NMR spectrum of homiamide A (**1**) in chloroform-*d*

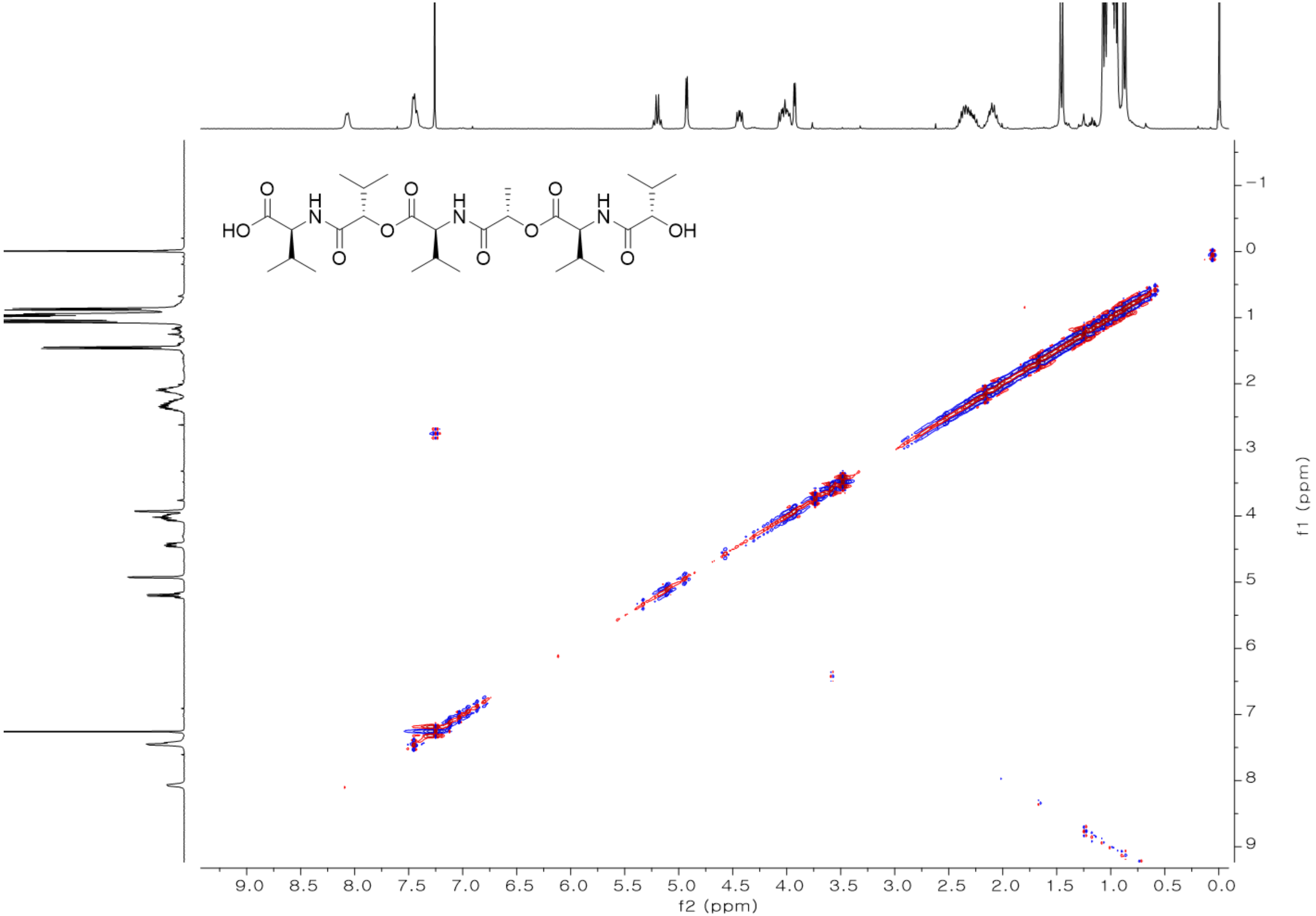

Figure S7. HRMS Spectrum for homiamide A (**1**)

[Mass Spectrum]
Data : FAB-E224 Date : 06-Dec-2022 17:07
RT : 0.81 min Scan# : (21,29)
Elements : C 100/0, H 100/0, N 5/0, O 15/5, Na 1/0
Mass Tolerance : 10ppm, 5mmu if m/z < 500, 10mmu if m/z > 1000
Unsaturation (U.S.) : -0.5 - 20.0

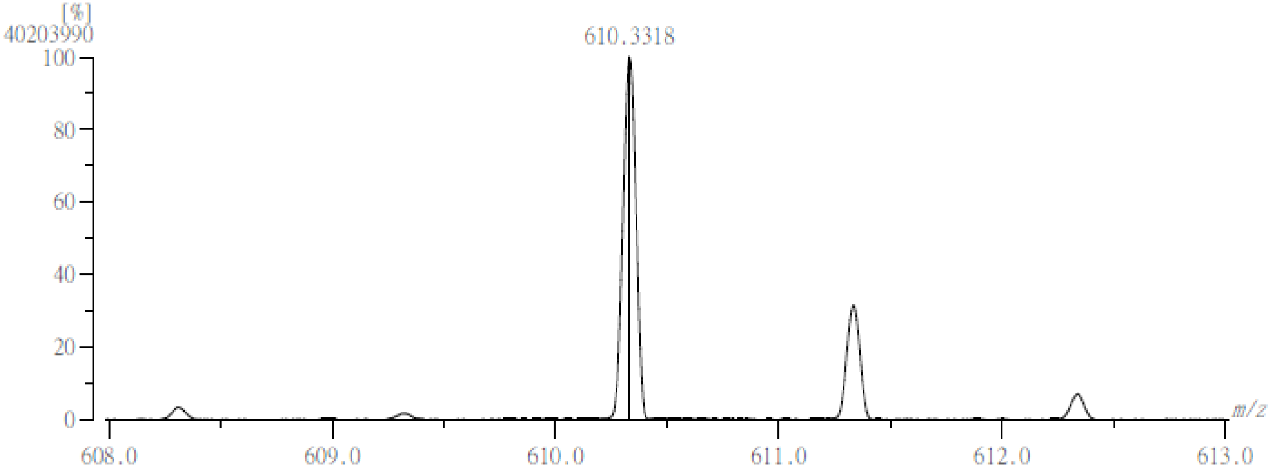

	Observed m/z	Int%	Err[ppm / mmu]	U.S.	Composition
1	610.3318	100.00	+6.1 / +3.7	17.5	C37 H44 N3 O5
2			+3.9 / +2.4	17.0	C39 H46 O6
3			-7.9 / -4.9	13.0	C33 H46 N4 O7
4			-3.6 / -2.2	8.5	C30 H48 N3 O10
5			-5.8 / -3.5	8.0	C32 H50 O11
6			+3.0 / +1.9	4.5	C25 H48 N5 O12
7			+0.8 / +0.5	4.0	C27 H50 N2 O13
8			+7.4 / +4.5	0.0	C22 H50 N4 O15
9			+7.8 / +4.8	14.0	C37 H47 O6 Na
10			-4.0 / -2.4	10.0	C31 H47 N4 O7 Na
11			-6.2 / -3.8	9.5	C33 H49 N O8 Na
12			+0.4 / +0.2	5.5	C28 H49 N3 O10 Na
13			-1.8 / -1.1	5.0	C30 H51 O11 Na
14			+7.0 / +4.3	1.5	C23 H49 N5 O12 Na
15			+4.8 / +2.9	1.0	C25 H51 N2 O13 Na

Figure S8. FT-IR spectrum of homiamide A (**1**)

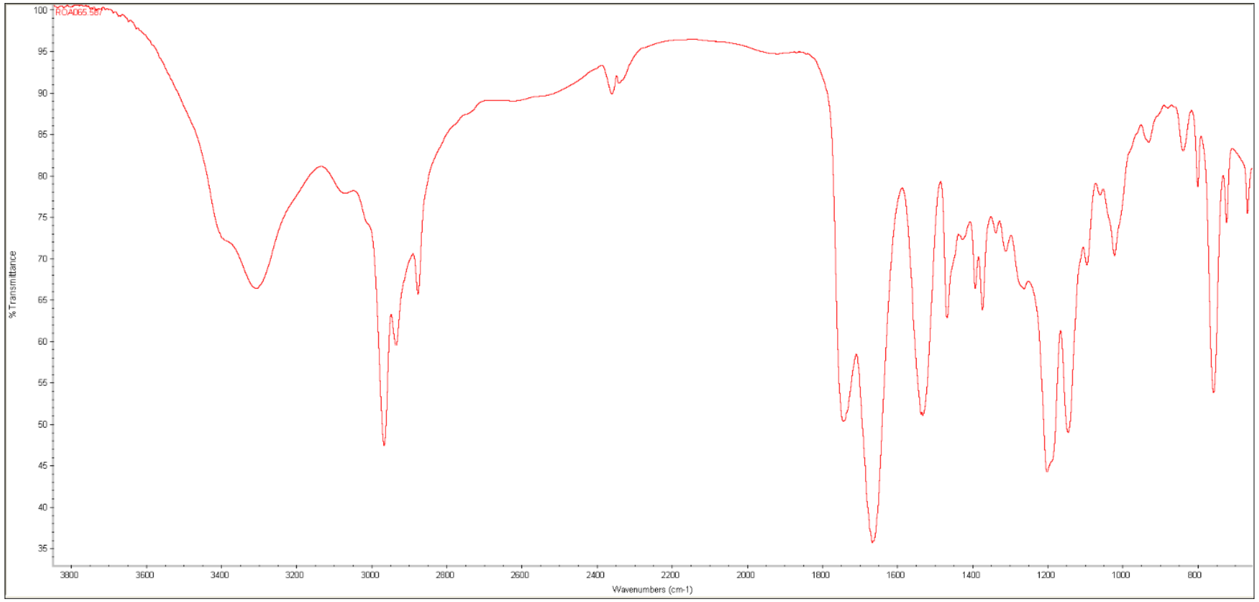

Figure S9. ^{1}H NMR spectrum of homiamide B (**2**) in chloroform-*d*

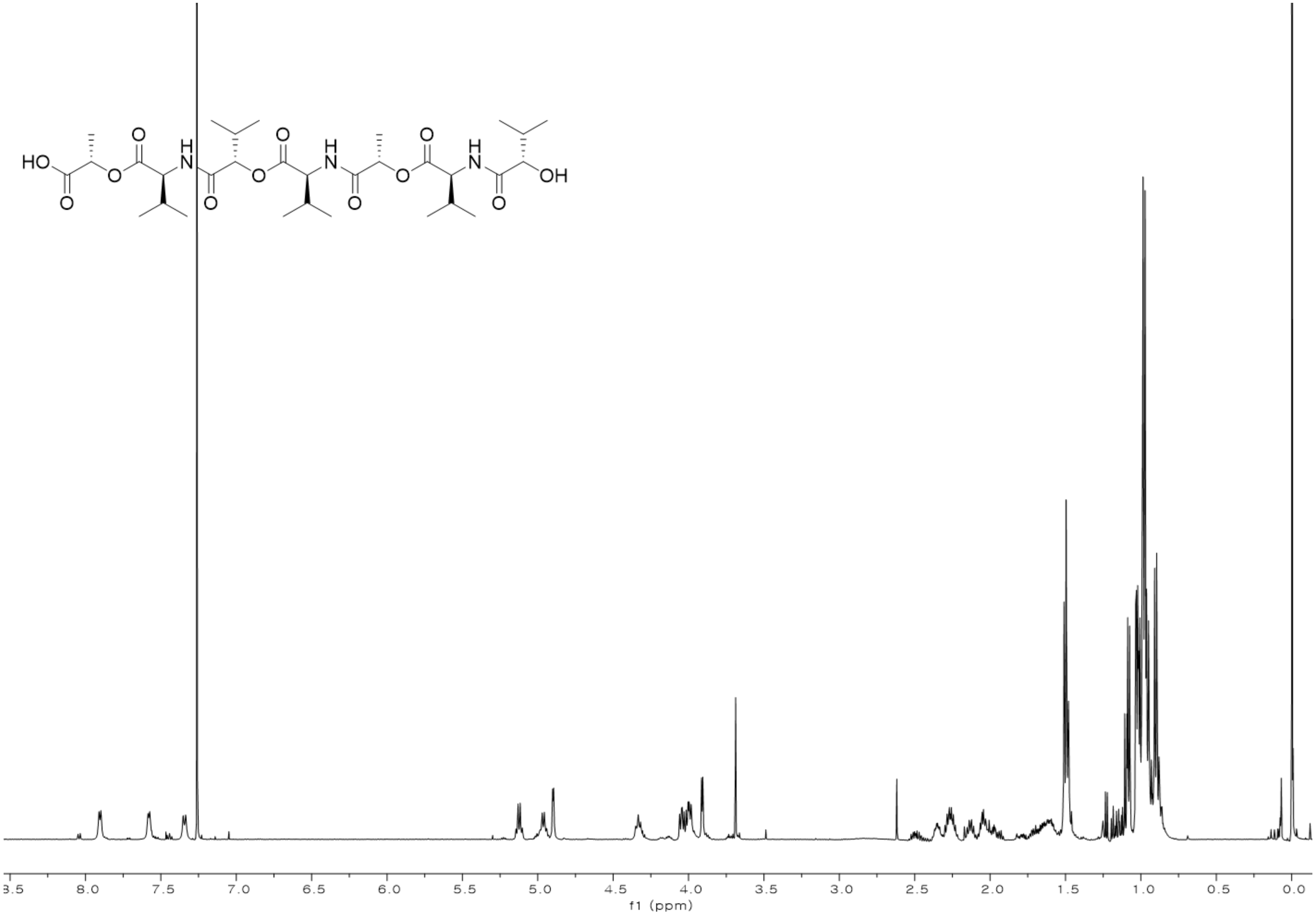

Figure S10. ^{13}C NMR spectrum of homiamide B (**2**) in chloroform-*d*

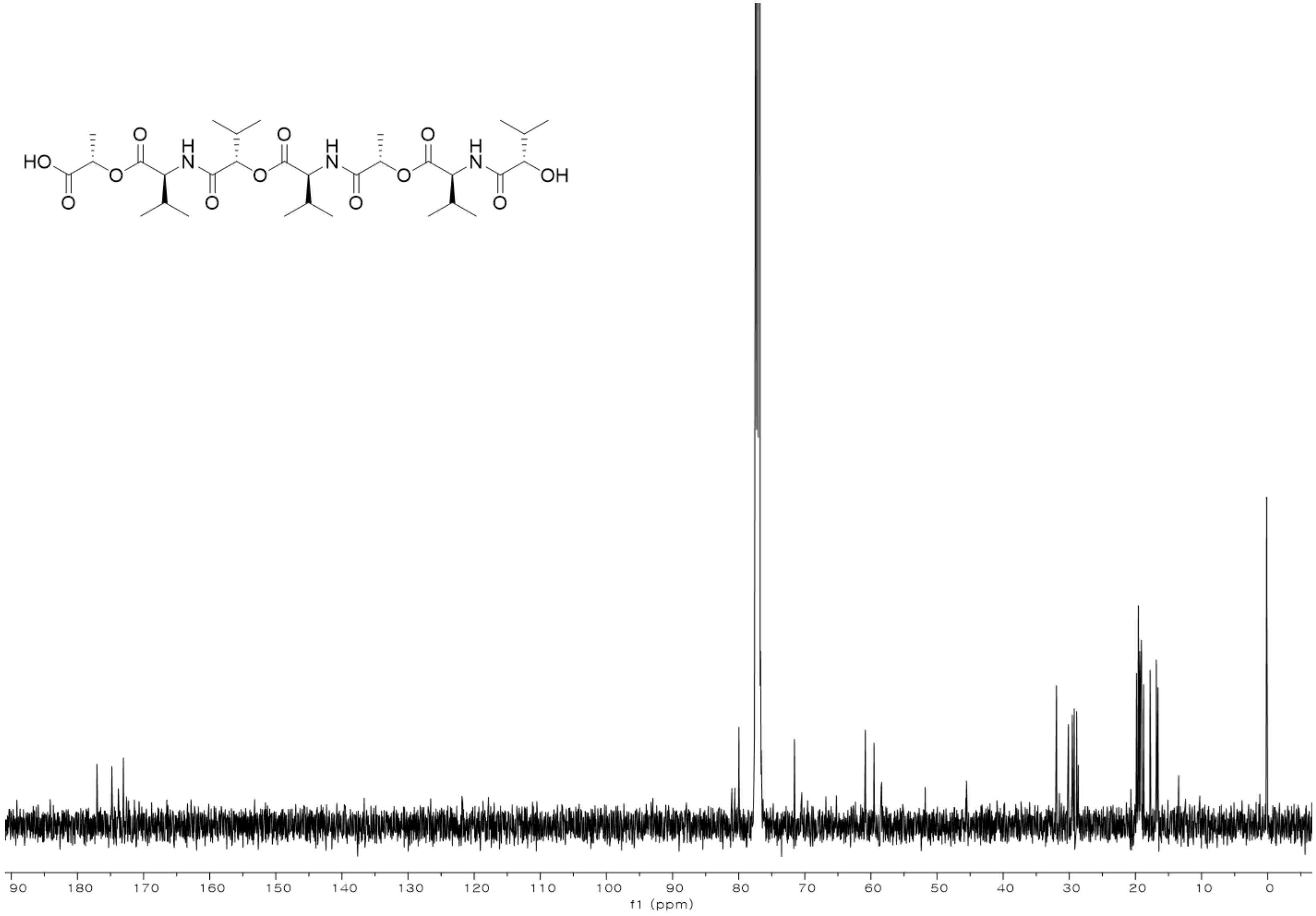

Figure S11. COSY spectrum of homiamide B (**2**) in chloroform-*d*

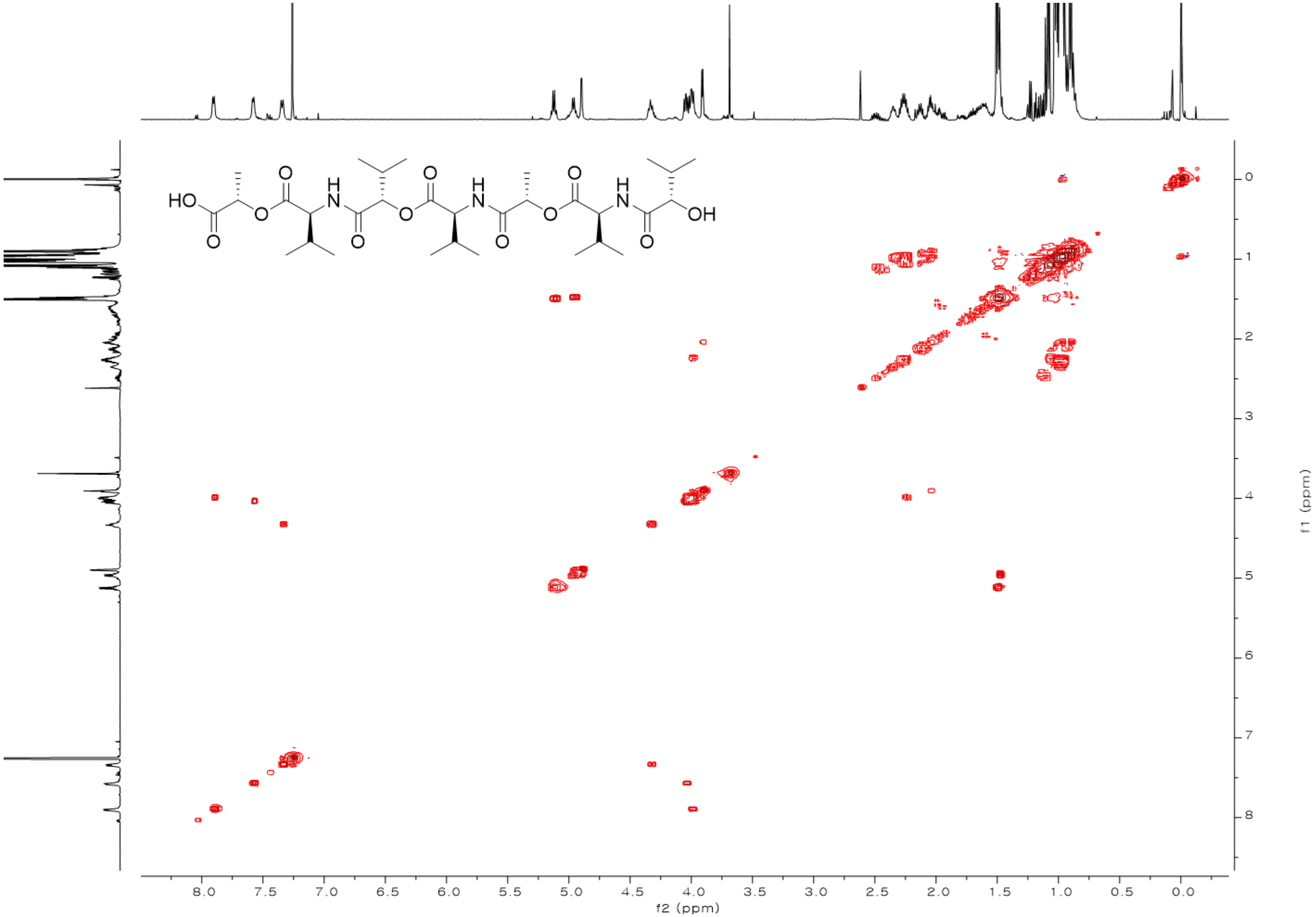

Figure S12. HSQC NMR spectrum of homiamide B (**2**) in chloroform-*d*

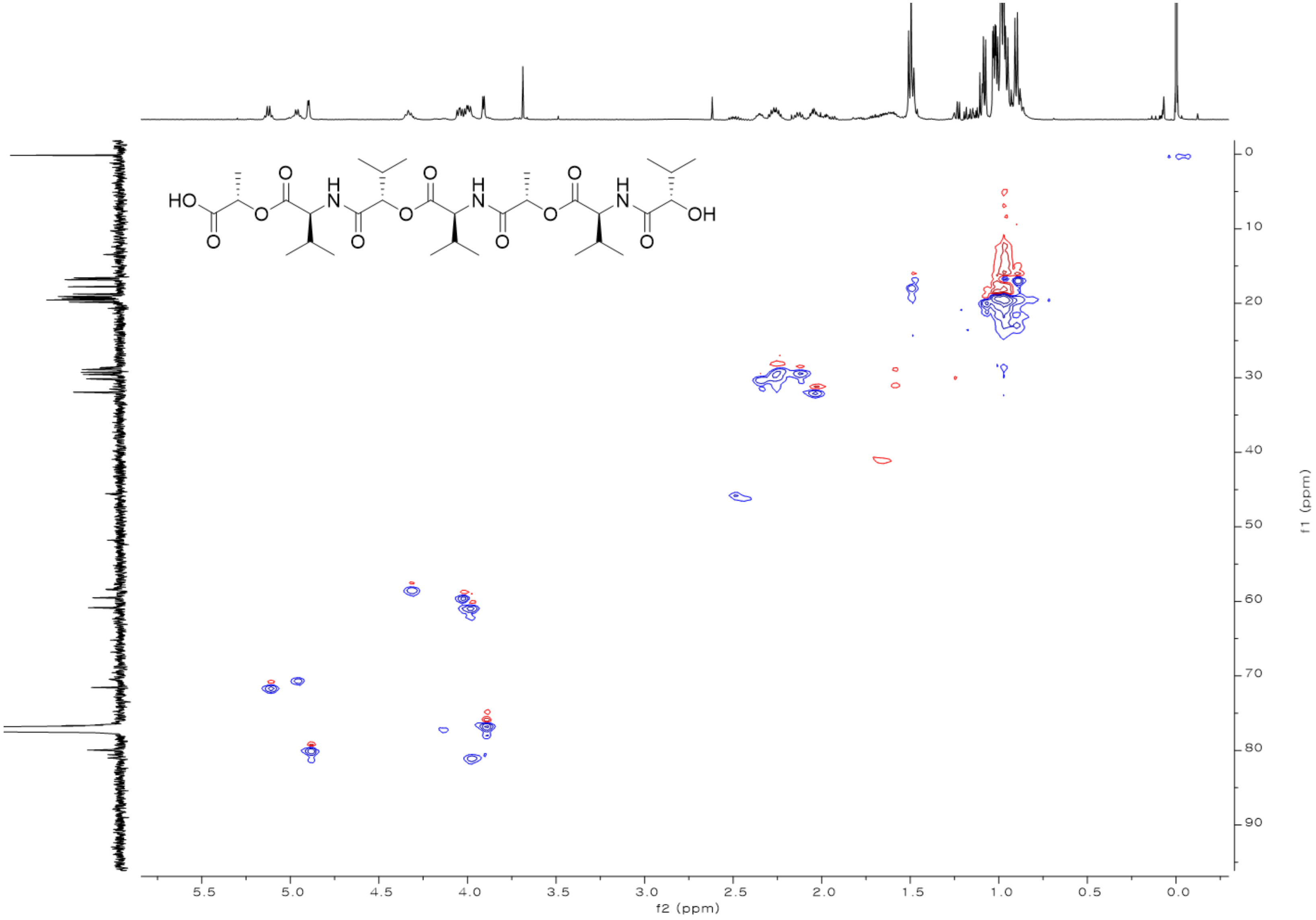

Figure S13. HMBC NMR spectrum of homiamide B (**2**) in chloroform-*d*

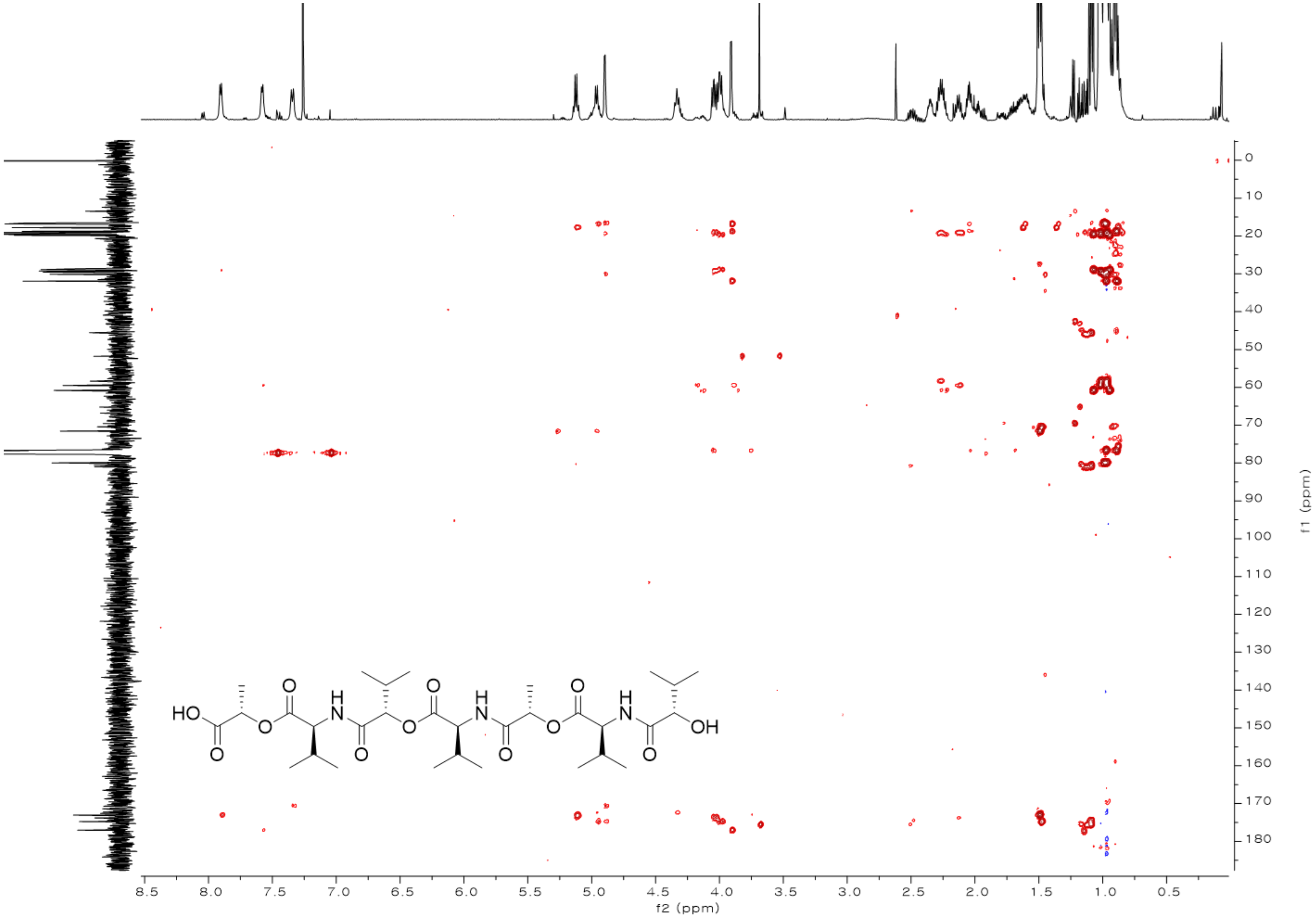

Figure S14. NOESY NMR spectrum of homiamide B (**2**) in chloroform-*d*

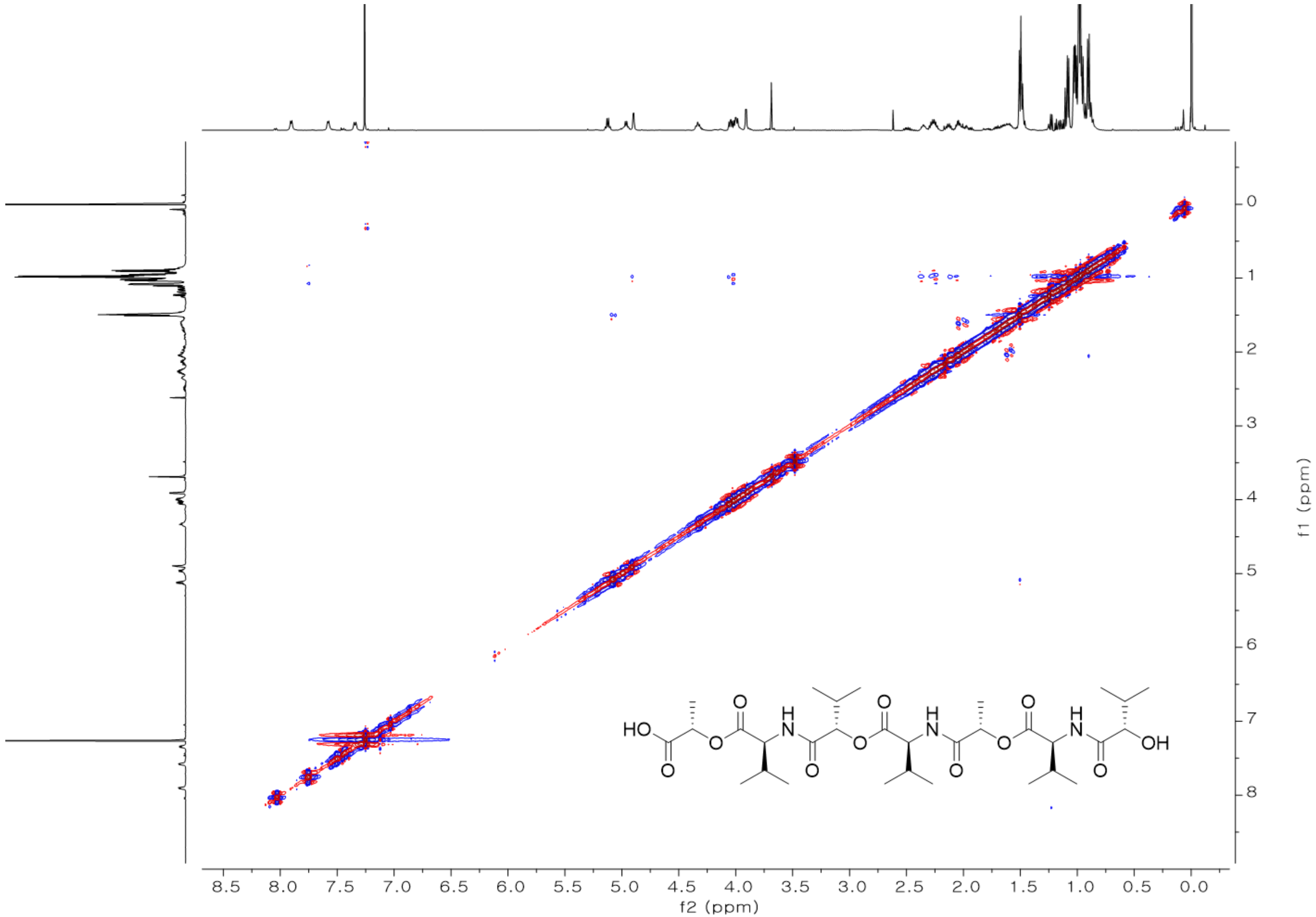

Figure S15. HRMS spectrum for homiamide B (**2**)

[Mass Spectrum]
Data : FAB-E223 Date : 06-Dec-2022 16:54
RT : 0.53 min Scan# : (15,39)
Elements : C 100/0, H 100/0, N 5/0, O 15/5, Na 1/0
Mass Tolerance : 10ppm, 5mmu if m/z < 500, 10mmu if m/z > 1000
Unsaturation (U.S.) : -0.5 - 10.0

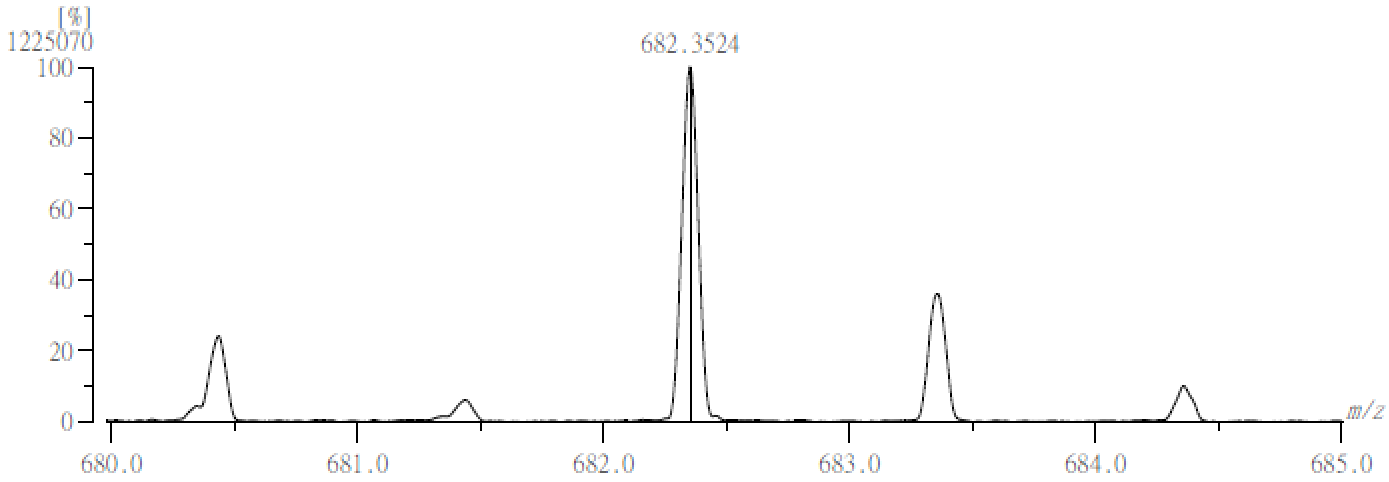

	Observed m/z	Int%	Err[ppm / mmu]	U.S.	Composition
1	682.3524	100.00	-4.0 / -2.7	9.5	C33 H52 N3 O12
2			-5.9 / -4.0	9.0	C35 H54 O13
3			+1.9 / +1.3	5.5	C28 H52 N5 O14
4			-0.0 / -0.0	5.0	C30 H54 N2 O15
5			-0.4 / -0.3	6.5	C31 H53 N3 O12 Na
6			-2.4 / -1.6	6.0	C33 H55 O13 Na
7			+5.5 / +3.7	2.5	C26 H53 N5 O14 Na
8			+3.5 / +2.4	2.0	C28 H55 N2 O15 Na

Figure S16. FT-IR spectrum for homiamide B (**2**)

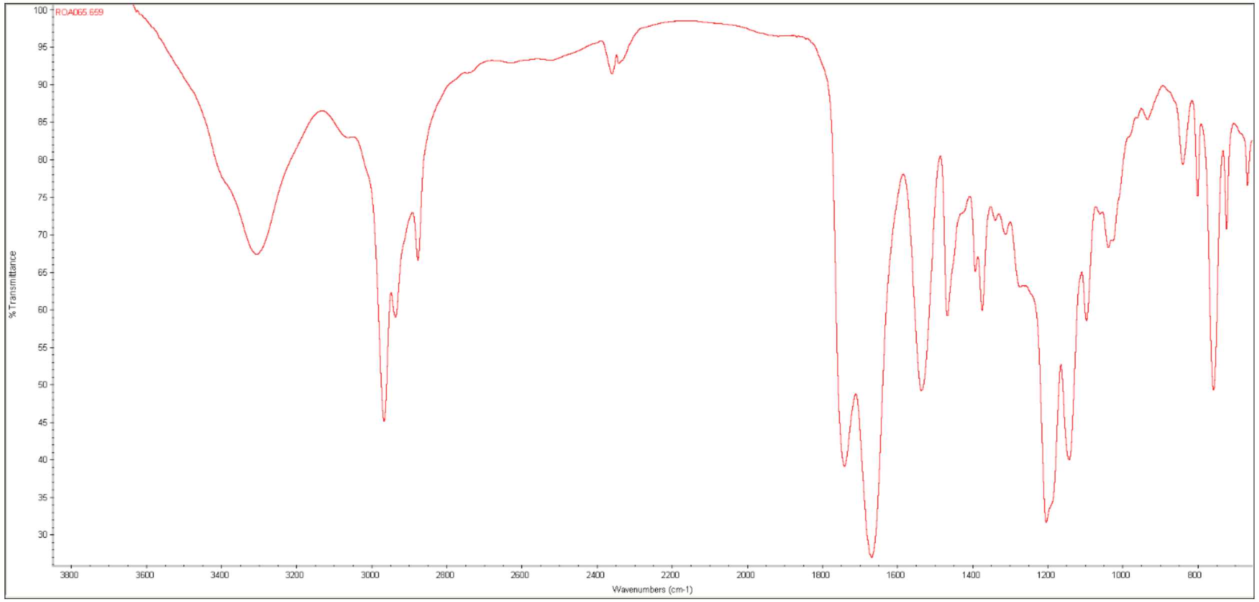

Figure S17. ^{1}H NMR spectrum of homiamide C (**3**) in chloroform-*d*

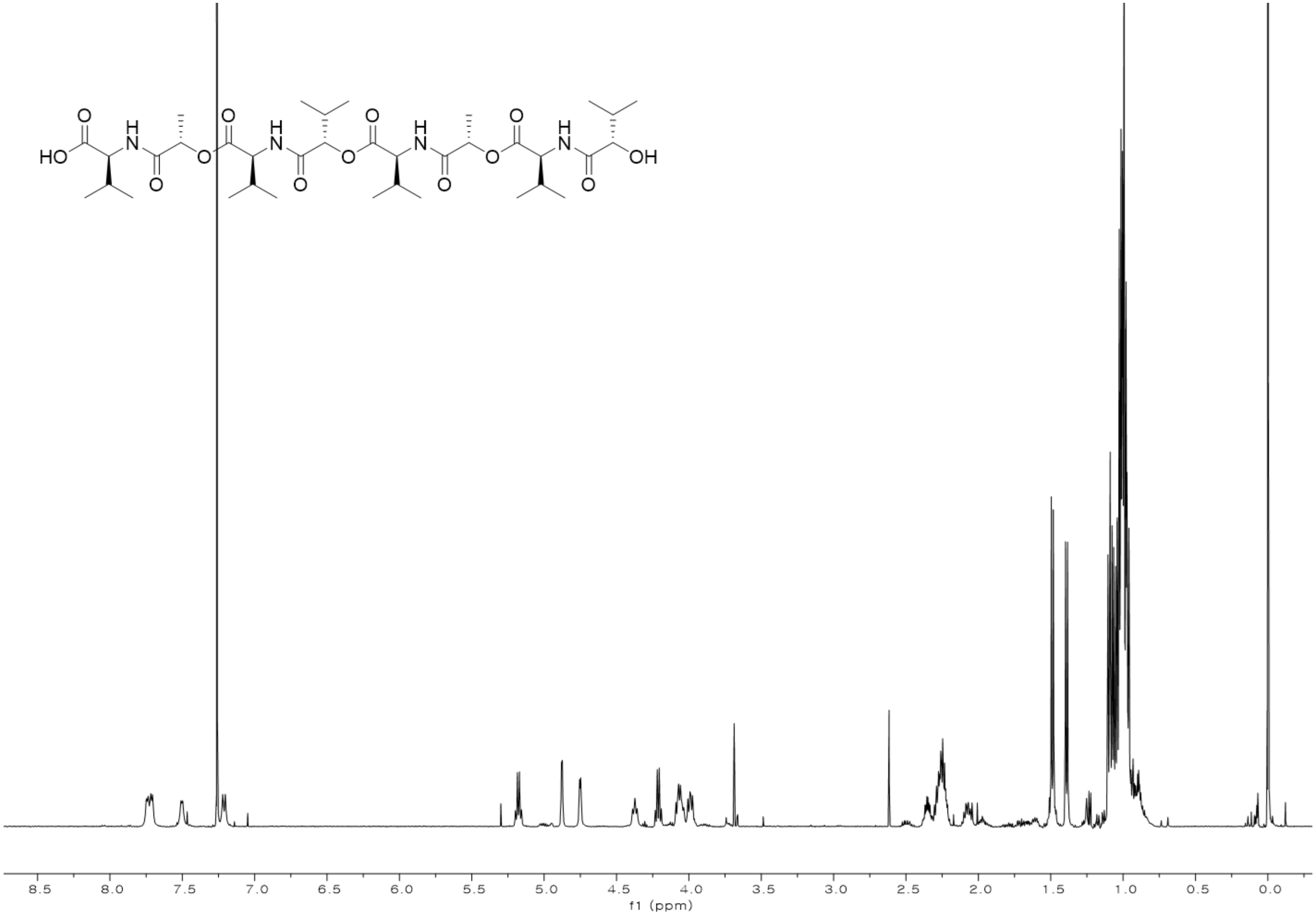

Figure S18. ^{13}C NMR spectrum of homiamide C (**3**) in chloroform-*d*

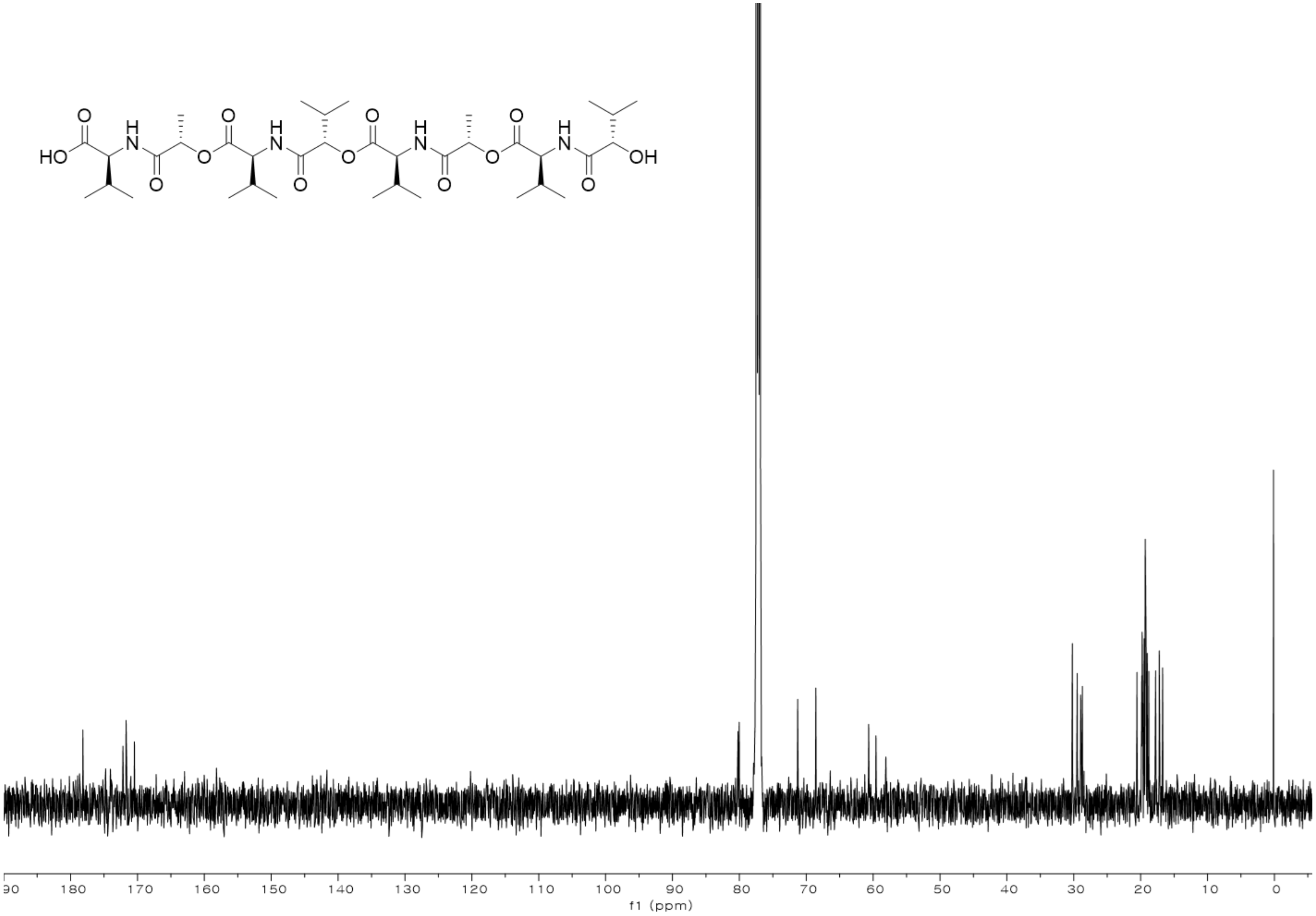

Figure S19. COSY spectrum of homiamide C (**3**) in chloroform-*d*

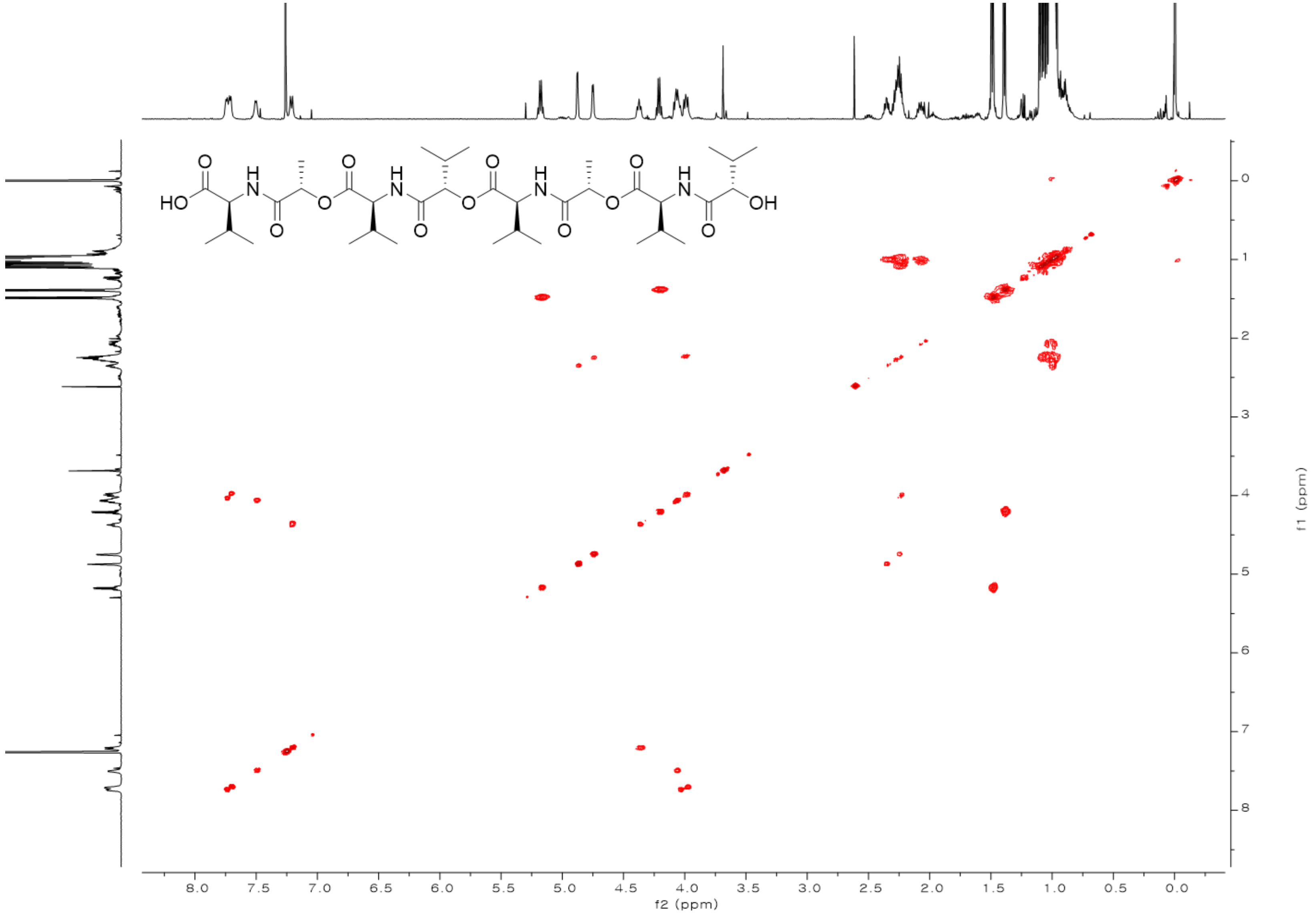

Figure S20. HSQC NMR spectrum of homiamide C (**3**) in chloroform-*d*

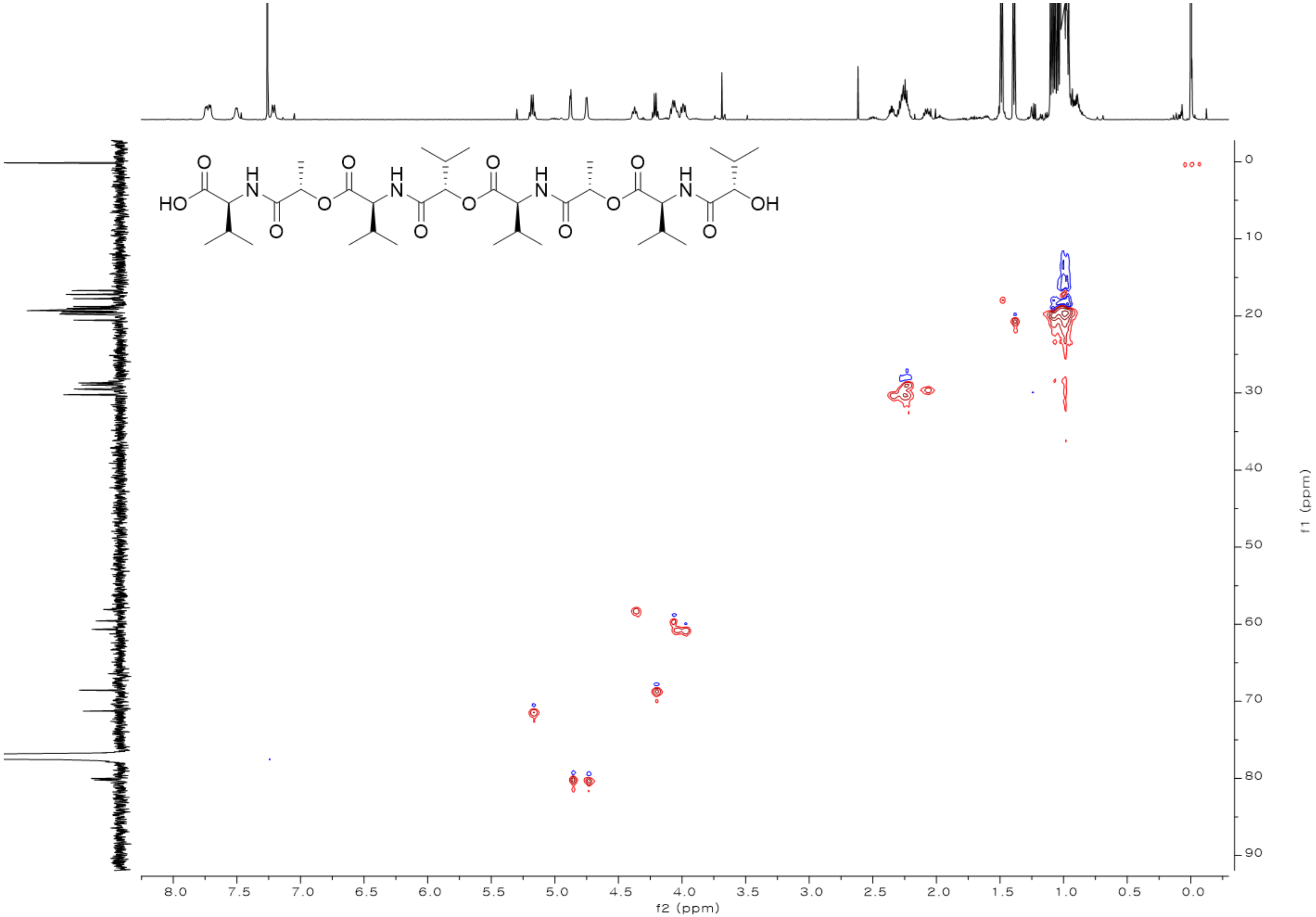

Figure S21. HMBC NMR spectrum of homiamide C (**3**) in chloroform-*d*

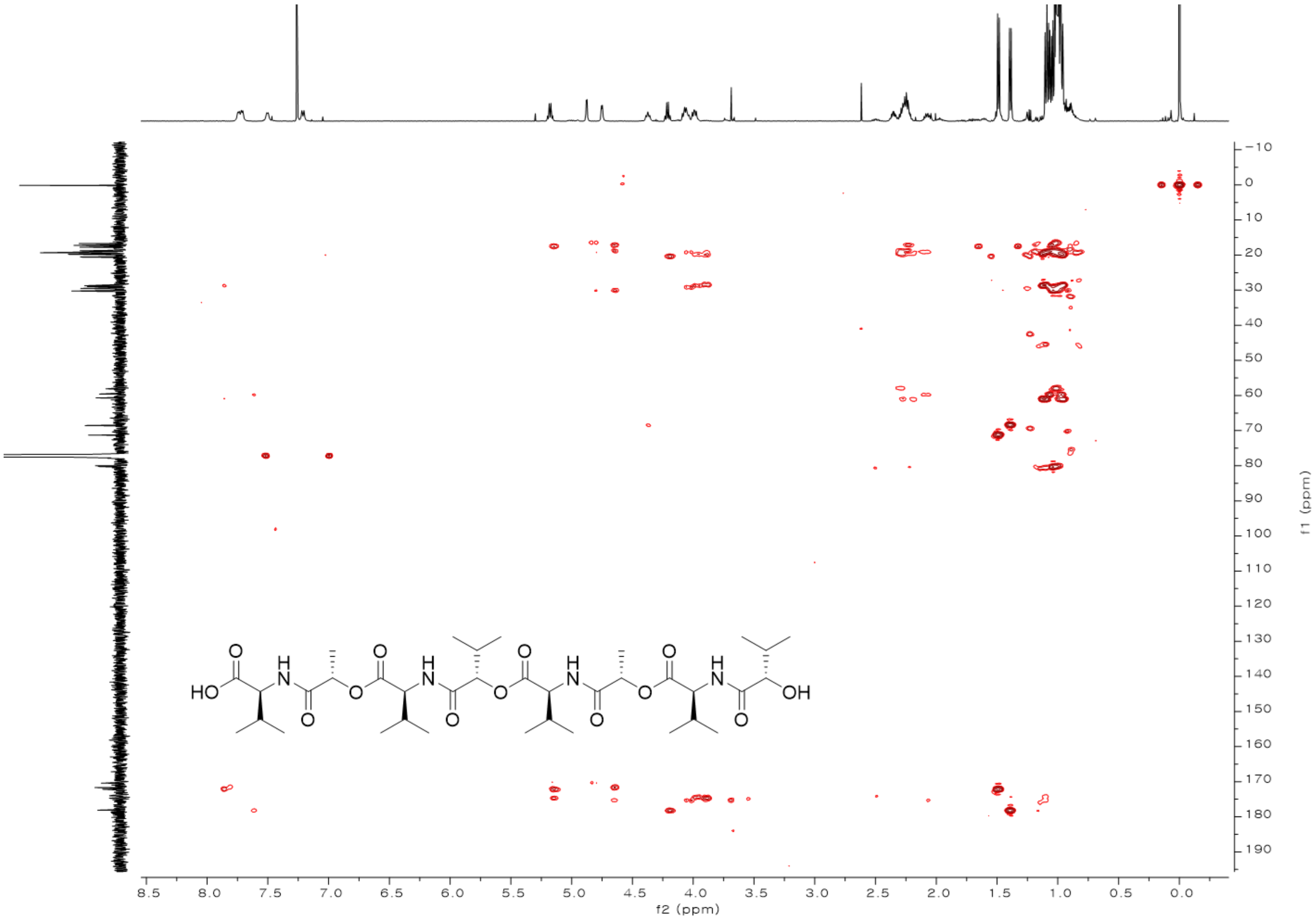

Figure S22. NOESY NMR spectrum of homiamide C (**3**) in chloroform-*d*

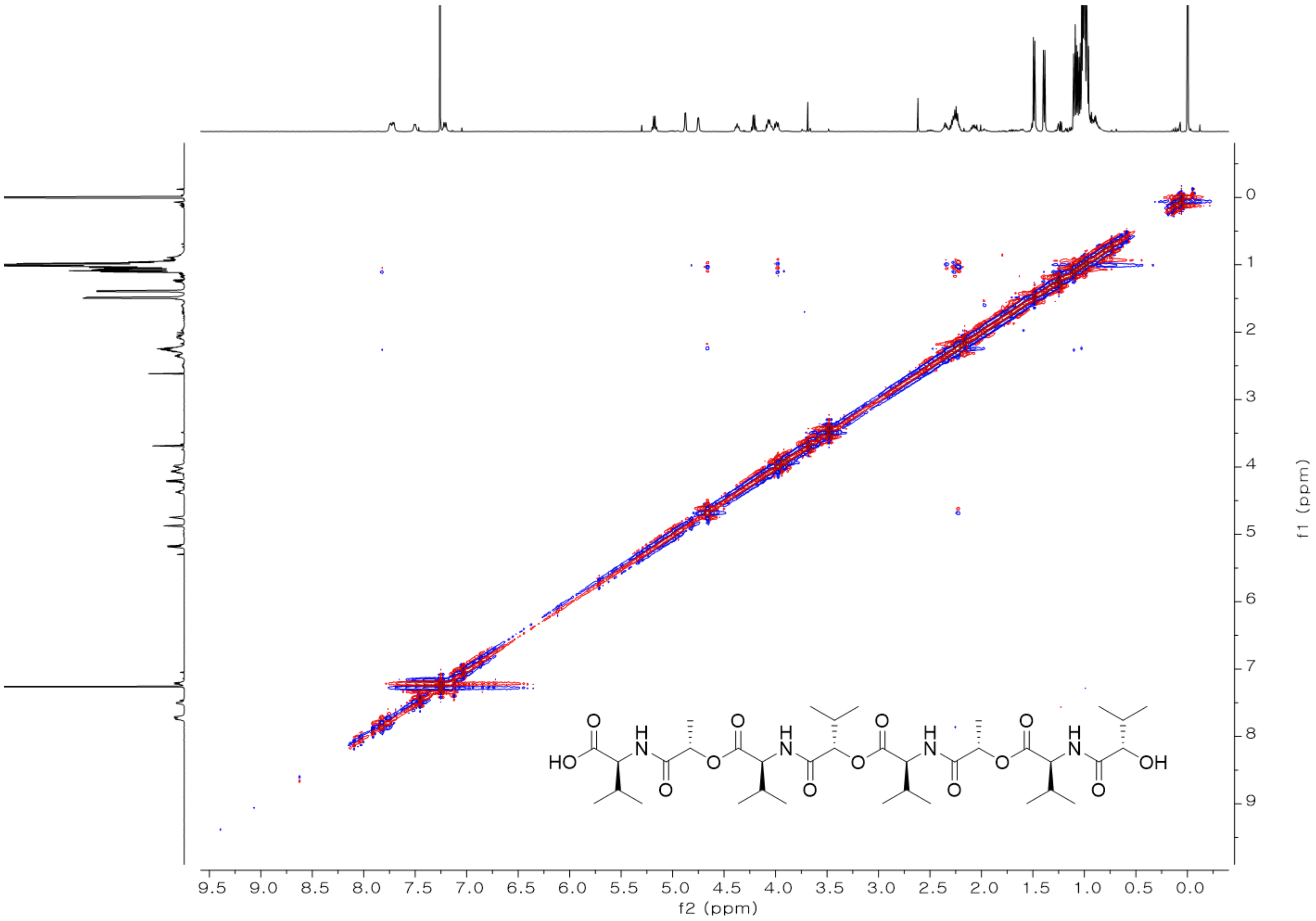

Figure S23. HRMS spectrum for homiamide C (**3**)

[Mass Spectrum]
Data : FAB-E220 Date : 05-Dec-2022 17:35
RT : 1.11 min Scan# : (33,37)
Elements : C 100/0, H 100/0, N 5/0, O 15/5, Na 1/0
Mass Tolerance : 10ppm, 5mmu if m/z < 500, 10mmu if m/z > 1000
Unsaturation (U.S.) : -0.5 - 20.0

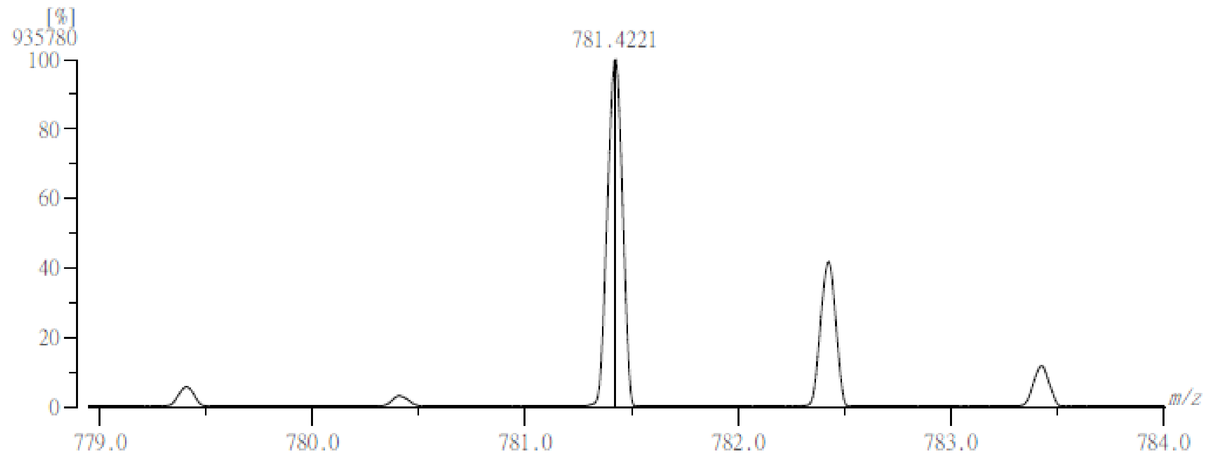

	Observed m/z	Int%	Err[ppm / mmu]	U.S.	Composition
1	781.4221	100.00	+5.7 / +4.5	19.5	C45 H57 N4 O8
2			+4.0 / +3.1	19.0	C47 H59 N O9
3			-5.2 / -4.1	15.0	C41 H59 N5 O10
4			-7.0 / -5.4	14.5	C43 H61 N2 O11
5			+9.1 / +7.1	15.0	C42 H59 N3 O11
6			+7.4 / +5.8	14.5	C44 H61 O12
7			-1.8 / -1.4	10.5	C38 H61 N4 O13
8			-3.5 / -2.8	10.0	C40 H63 N O14
9			-7.3 / -5.7	16.0	C44 H60 N3 O8 Na
10			+8.8 / +6.9	16.5	C43 H58 N4 O8 Na
11			-9.0 / -7.1	15.5	C46 H62 O9 Na
12			+7.1 / +5.5	16.0	C45 H60 N O9 Na
13			-2.2 / -1.7	12.0	C39 H60 N5 O10 Na
14			-3.9 / -3.0	11.5	C41 H62 N2 O11 Na
15			+1.3 / +1.0	7.5	C36 H62 N4 O13 Na
16			-0.4 / -0.4	7.0	C38 H64 N O14 Na
17			-9.7 / -7.6	3.0	C32 H64 N5 O15 Na

Figure S24. FT-IR spectrum for homiamide C (**3**)

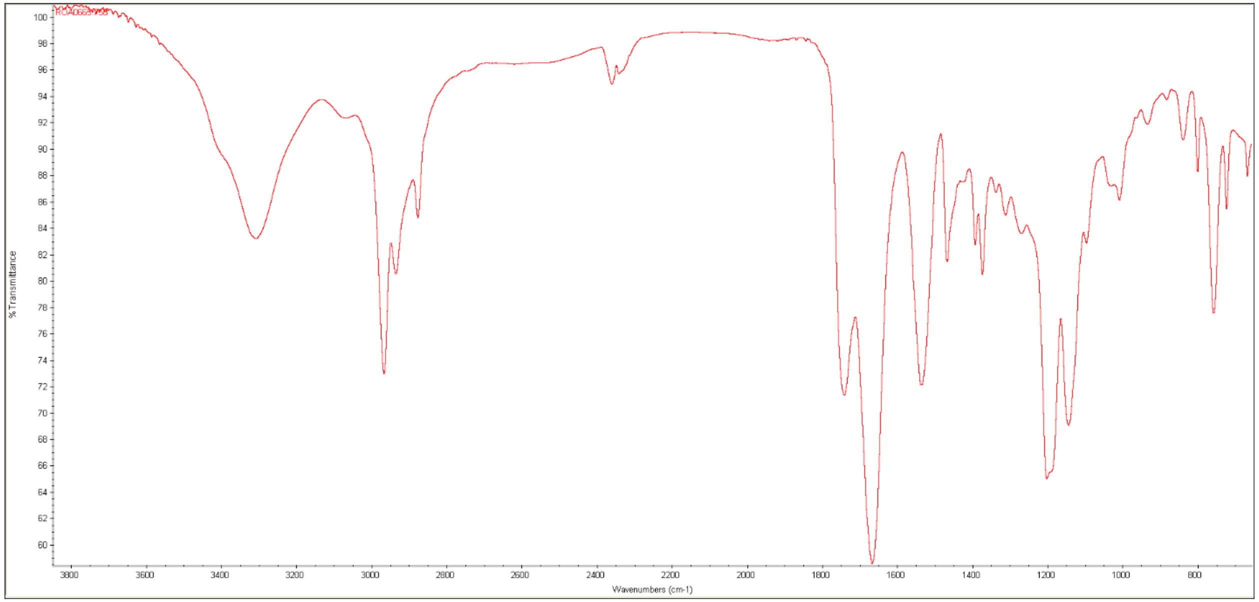

Figure S25. ^{1}H NMR spectrum of AI-77-C (**4**) in methanol-d_4

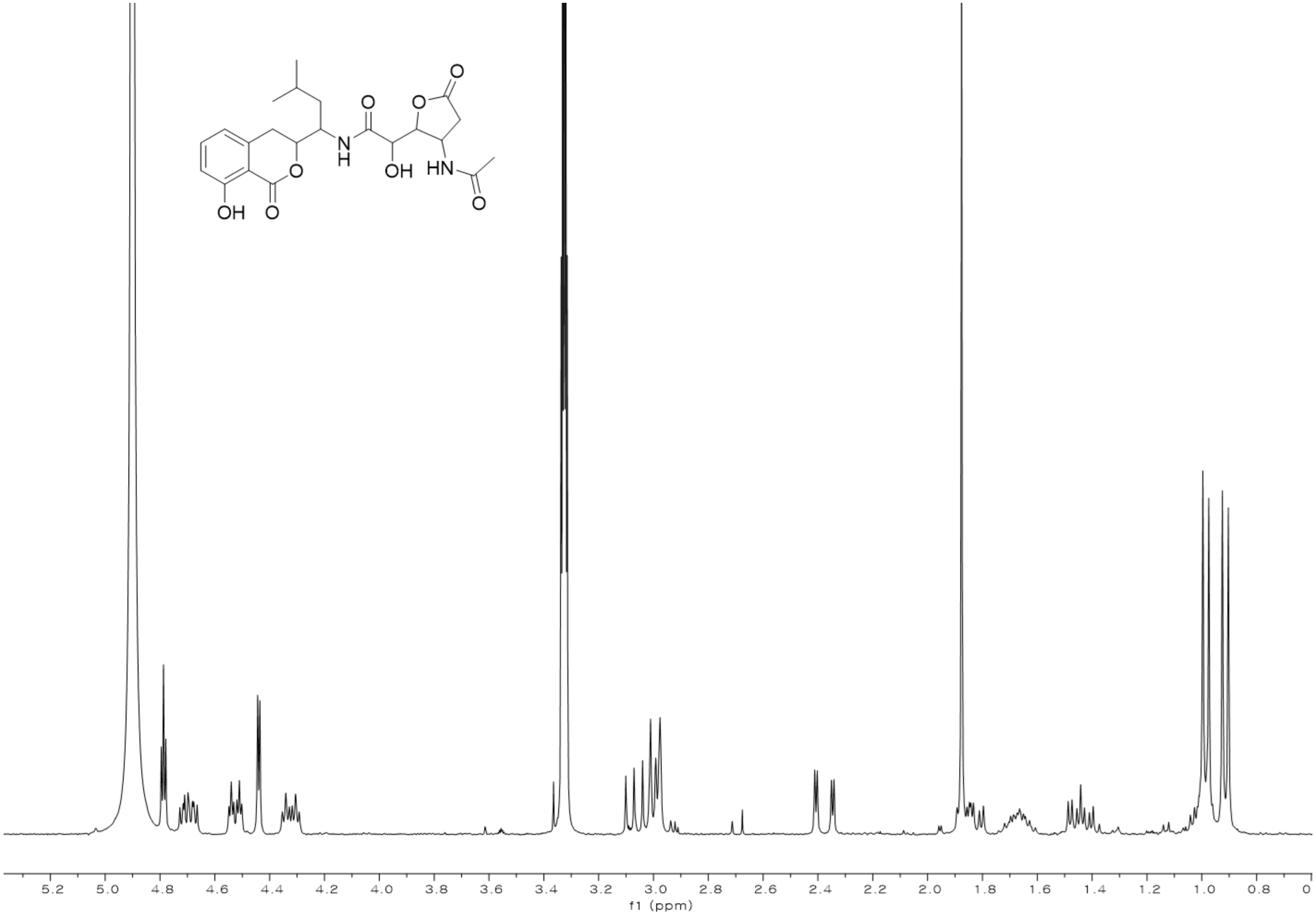

Figure S26. LC chromatograms of L- and D-FDLA derivatives of Valine

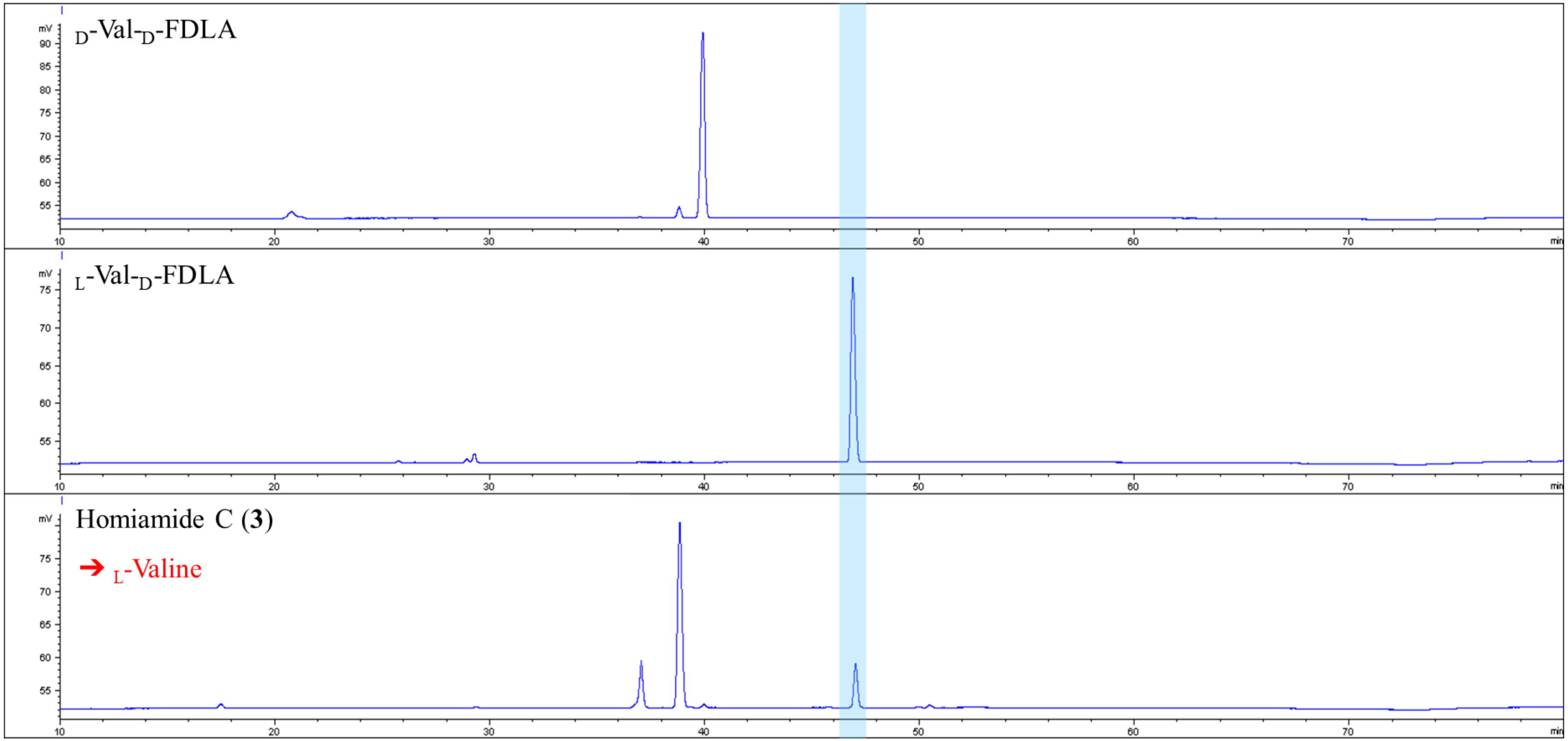

Figure S27. Chromatograms of Lac and Hiv in **(a)** homiamide A (**1**) and **(b)** homiamide C (**3**) as tri-methylsilyl derivative

(a)

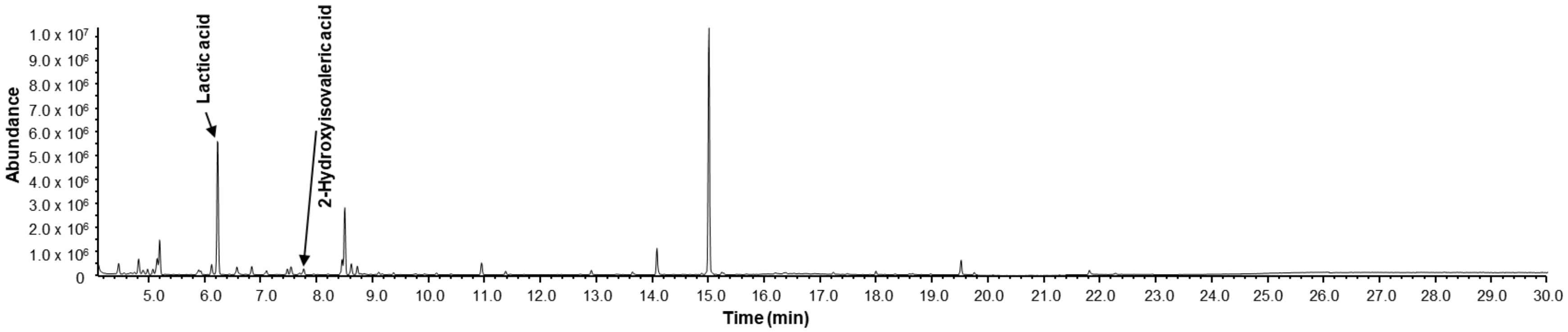

(b)

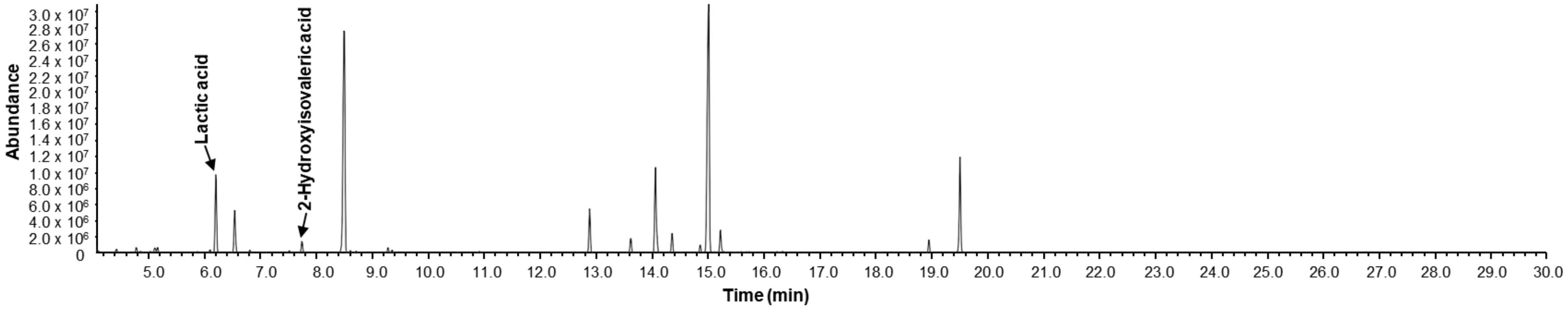

Figure S28. GC-MS analysis of *O*-trifluoroacetylated (*S*)-(+)-3-methyl-2-butyl ester of Lac and Hiv standards **(a)** SIM chromatogram **(b)** Expanded SIM chromatogram **(c)** Selected electron ionization mass spectrum of Lac and Hiv

(a)

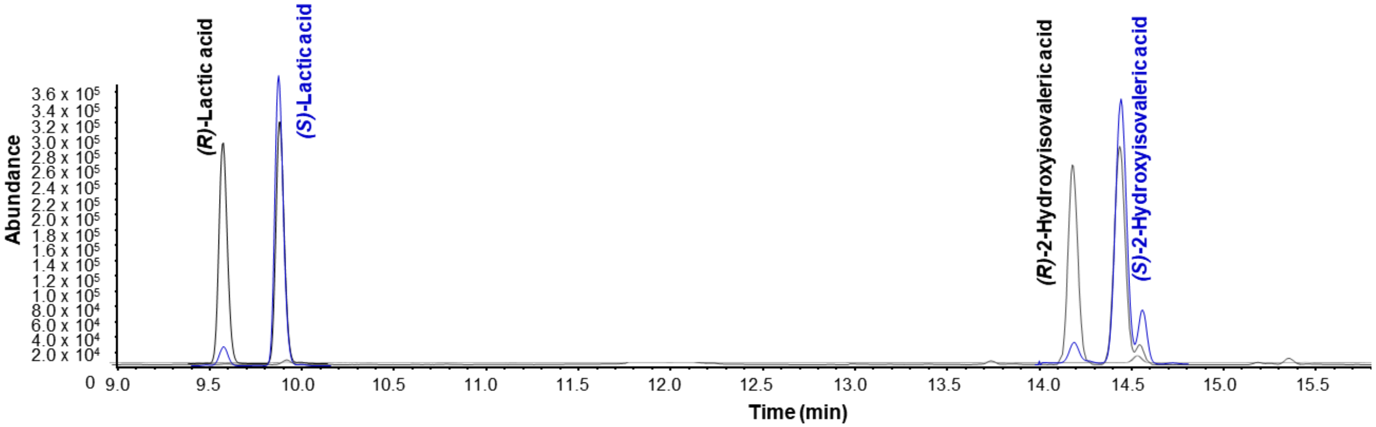

(b)

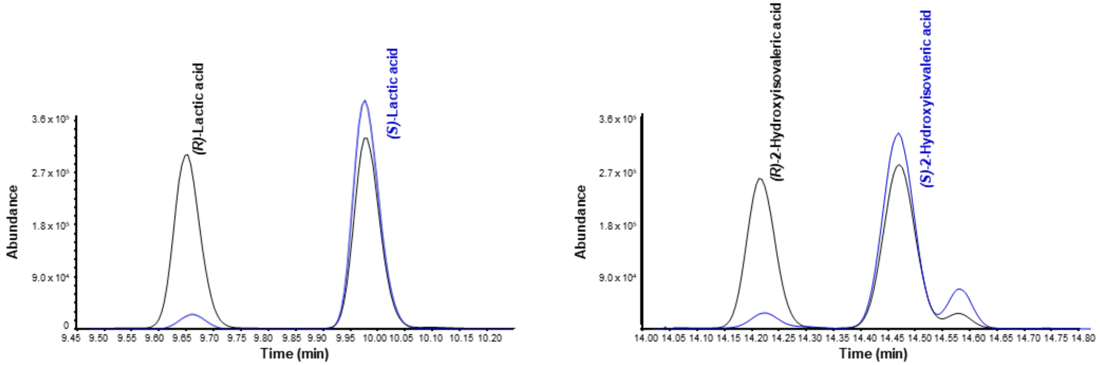

(c)

	Lac	Hiv
Selected ion	**169,** 213, 241	**197**, 241, 289

Figure S29. SIM Chromatograms for *O*-trifluoroacetylated (*S*)-(+)-3-methyl-2-butyl ester of Lac and Hiv enantiomers in **(a)** homiamide A (**1**) and **(b)** homiamide C (**3**)

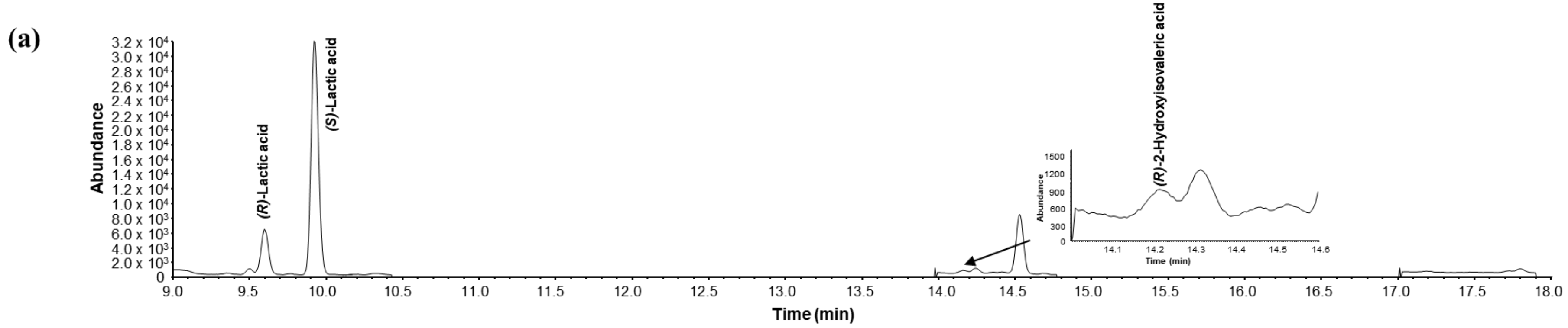

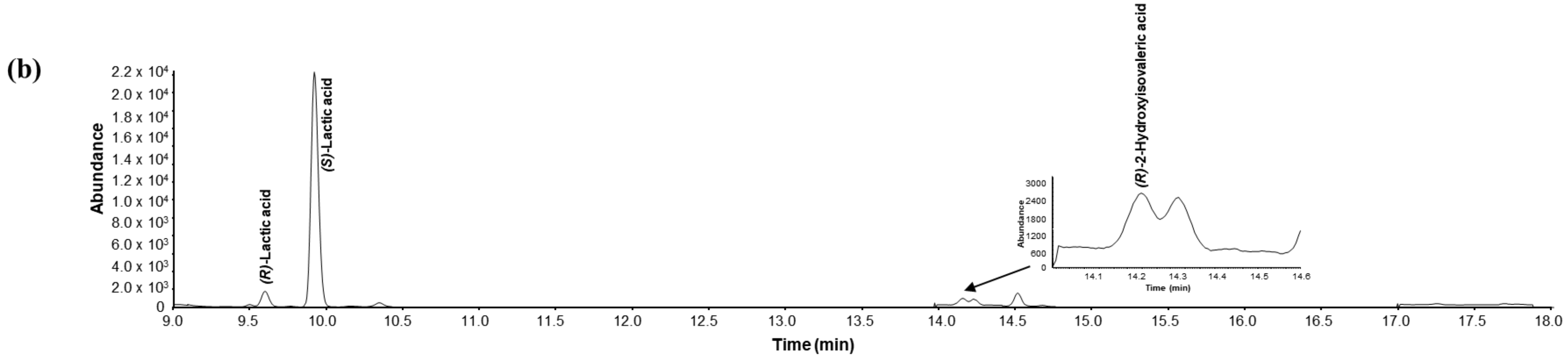

Supplement: Supplementary file 1 [file molecules-29-05539-s001.zip › molecules-3309607-supplementary.pdf]
